# Direct measurement of Criegee intermediates in isoprene ozonolysis

**DOI:** 10.1038/s41467-026-73307-6

**Published:** 2026-05-20

**Authors:** Lei Yang, Katia Hatem, Mixtli Campos-Pineda, Jingsong Zhang

**Affiliations:** 1 Department of Chemistry, University of California, Riverside, CA USA; 2 Air Pollution Research Center, University of California, Riverside, CA USA; 3 Present Address: Centre for Research into Atmospheric Chemistry, University College Cork, Cork, Ireland

**Keywords:** Reaction kinetics and dynamics, Atmospheric chemistry, Optical spectroscopy

## Abstract

Isoprene is the most abundant unsaturated hydrocarbon in the troposphere. Carbonyl oxides, also called Criegee intermediates, are highly reactive transient species in the ozonolysis of alkenes that play important roles in tropospheric oxidation. Despite decades of efforts, direct observation of Criegee intermediates in isoprene ozonolysis has not been achieved. Here we show the first direct measurement of Criegee intermediates produced in isoprene ozonolysis. Ultra-violet spectra of Criegee intermediates are captured with cavity ringdown spectroscopy and match oscillatory π* ← π transitions of CH_2_OO. The in-situ concentration time profiles of Criegee intermediates allow direct kinetic studies and benchmark the isoprene ozonolysis reaction network. The results indicate that CH_2_OO dominates stabilized Criegee intermediate chemistry in the low-pressure region, while the role of larger stabilized four-carbon Criegee intermediates could be important in tropospheric oxidation.

## Abstract (web summary)

Isoprene dominates tropospheric unsaturated hydrocarbons. This work reports the direct detection of Criegee intermediates in isoprene ozonolysis, resolving a persistent challenge and providing key constraints on atmospheric oxidation chemistry.

## Introduction

Isoprene, a conjugated diene (C_5_H_8_), is the dominant volatile organic compound (VOC) in the atmosphere, accounting for nearly half of all nonmethane VOC emissions, with a global flux exceeding 500 Tg per year^1–10^. Through its oxidation by hydroxyl radicals (OH), ozone (O_3_), and nitrogen oxides (NO_x_), isoprene strongly influences the oxidizing capacity of the troposphere and contributes substantially to the formation of secondary organic aerosol (SOA)^1–7,11–13^. The reaction of isoprene with ozone, termed ozonolysis, is its second most important atmospheric sink (after OH), accounting for approximately 10% of its global loss^6,14–17^.

**Fig. 1:**
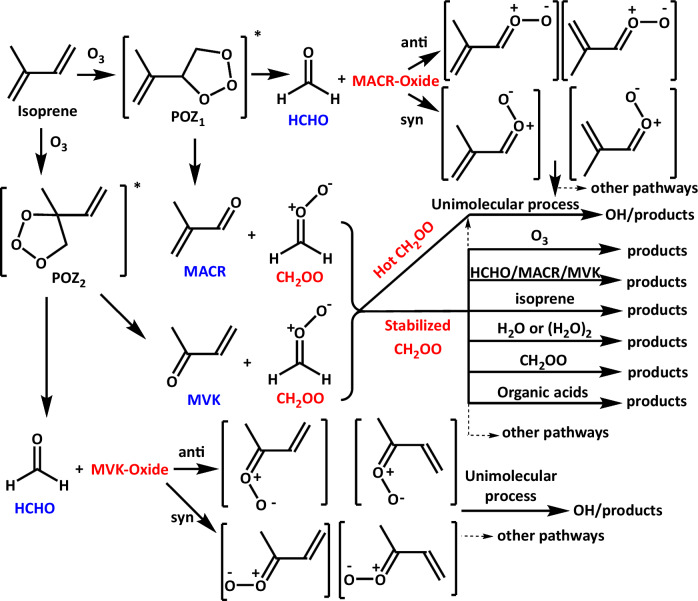
The formation and main consumption pathways of CIs (red) and carbonyls (blue) in ozonolysis of isoprene^6,17,60^.

Criegee intermediates (CIs), highly reactive zwitterionic carbonyl oxides named after Rudolf Criegee^18^, are central to the atmospheric impact of alkene ozonolysis^19–26^. These reactive intermediates initiate OH or organic radical chemistry^22,27,28^, oxidize trace gases such as SO_2_ and NO_2_^19,21^, and promote the formation of SOA^2–7,11^. Their importance has inspired numerous experimental and theoretical works, especially in the last decade, after Welz et al.^19^ developed the synthetic method of gas-phase CIs based on photolysis of diiodoalkanes in O_2_, which enabled the generation of stabilized CIs (sCIs) with low internal energy for fundamental studies aimed at unraveling their structure, spectroscopic characteristics, kinetics, and decomposition dynamics under laboratory conditions^24,25,29–35^. In the atmosphere, however, CIs are generated through 1,3-dipolar cycloaddition of ozone to alkenes, followed by rapid decomposition of the primary ozonides (POZs)^32^. For isoprene, this reaction is believed to proceed via multiple POZ pathways due to its conjugated diene structure, yielding a complex array of CIs, including CH_2_OO and four-carbon (C_4_) CIs such as methyl-vinyl-ketone (MVK) oxide and methacrolein (MACR) oxide, as shown in Fig. 1^6^ and Supplementary Fig. 1. CIs are often formed with broad energy distributions: hot CIs with excess internal energy (ΔE ≈ − 50 kcal mol^−1^ from POZ decomposition^36^) undergo prompt unimolecular decomposition or collisional stabilization^37^, while sCIs can react with atmospheric species such as SO_2_, NO_2_, and water, contributing to SOA nucleation and growth^30^.

Despite the central role of CIs in the atmosphere, their direct detection in ozonolysis conditions remains elusive^38,39^. The transient nature and chemical complexity of isoprene-derived CIs pose major challenges for direct experimental observation and accurate representation in their atmospheric chemical transport models. Current experimental investigations of CIs rely on the surrogate generation technique by photolysis of diiodoalkanes in O_2_^15,19,40^, but these do not replicate the energetic and chemical complexity of the alkene ozonolysis reaction network and cannot probe the nascent reaction dynamics and internal energy distributions of CIs. In addition, the yield of sCIs, their branching ratios, and major reaction pathways can only be determined in actual ozonolysis. In the case of isoprene, no study to date has directly observed CIs as a product of its ozonolysis^41^, leaving a fundamental gap in both mechanistic understanding and atmospheric modeling of CI formation and fate. Despite extensive efforts of CI studies^19–23,27,42–44^, some fundamental questions remain regarding their direct characterization in ozonolysis reactions, their lifetimes under atmospheric conditions, and the extent to which they influence tropospheric chemistry. Environmental chamber studies have offered valuable indirect insights into the sCI chemistry by monitoring product formation from reactions with scavengers such as SO_2_^17^, but interpretation of these results depends strongly on kinetic modeling assumptions and structure of the underlying reaction network (Supplementary Fig. 1). Direct detection of CIs, in contrast, enables explicit probing of their formation and fate, bridging the gap between isolated kinetic studies and the complex implementation of reaction mechanisms in environmental chambers^38^. In situ time-resolved measurements of key intermediates provide critical benchmarks for evaluating the completeness and accuracy of ozonolysis reaction networks and their application in atmospheric models^38^.

In this work, we present the direct detection of CIs formed from the ozonolysis of isoprene under controlled laboratory conditions. Using near-UV cavity ring-down spectroscopy (CRDS), we capture the characteristic absorption features of CH_2_OO. The selective CI titrant, SO_2_, is added as an effective scavenger to further confirm that these features belong to CIs. This work provides unequivocal experimental support for the formation of CIs in isoprene ozonolysis and establishes a pathway for probing the branching and reactivity of sCIs in the complex multichannel isoprene ozonolysis system, addressing a longstanding gap between atmospheric observations and mechanistic models. The results show that the nascent stabilization yield of smaller CIs (about 21% for CH_2_OO) is much higher than that of larger CIs (near zero, due to energy partitioning between CIs and byproduct carbonyls, as detailed in discussion), indicating that sCIs in the low-pressure region are mainly composed of CH_2_OO, while larger C_4_ CIs (MVK-oxide and MACR-oxide) are born with high energy and tend to undergo unimolecular isomerization or decompositions promptly. However, in the troposphere at the atmospheric pressure, when collisional stabilization is significant, C_4_ sCIs may also play an important role.

## Results

**Fig. 2:**
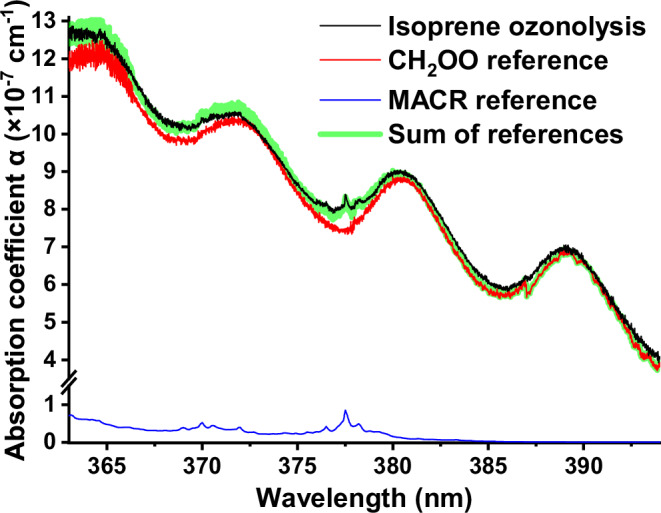
UV absorption spectra of isoprene ozonolysis (black) compared to vibronic reference spectra of CH_2_OO (red) and MACR (blue), and the sum of the two references (green). Determination of the concentrations of CH_2_OO (1.2 × 10^11 ^cm^−3^) and MACR (2.6 × 10^12 ^cm^−3^) is by fitting and scaling corresponding absorption cross-section data by Campos-Pineda et al.^38,45^ and Meller et al.^52,64^, respectively. Contributions from MVK, HCHO, and O_3_ (or other species) are not included in the fitting because their absorptions in the 363–394 nm range are small (less than MACR) and not discernible. Source data are provided as a Source Data file.

Figure 2 shows the high-resolution absorption spectra of isoprene ozonolysis (black) and comparison to reference spectra of CH_2_OO (red), scaled from its absorption cross sections measured in ethene ozonolysis by Campos-Pineda et al.^38^ In this experiment, isoprene (initial concentration of 5 × 10^15 ^cm^−3^) was mixed with O_3_ (9.3 × 10^14 ^cm^−3^) in the fast flow reactor at a residence time of 0.15 s at 7.5 Torr and 293 K while CRDS recorded the near-UV spectra in situ in the 363−394 nm range, as detailed in the Supplementary Materials and Supplementary Fig. 2. The broad spectral features spaced by 8 nm with a half-peak width of 3.5 nm correspond to the B̃(^1^A′) ← X̃(^1^A′) vibronic bands of CH_2_OO^38,45^, attributed to the π* ← π electron transition localized on the C–O–O carbonyl group^33,46^. The short residence time minimized the production of byproducts, enhancing the absorption signal of nascent stabilized CH_2_OO with little interference from coproducts, except for a small broad absorption with several diffuse vibronic bands (at ~370 and ~377.5 nm) due to MACR, as shown in the MACR reference (blue). The concentration of CH_2_OO in the representative spectra (black) is determined from fitting with the reference spectra to be 1.2 × 10^11 ^cm^−3^ (see details in the Experimental Methods in Supplementary Materials). As compared in Supplementary Fig. 3, cross sections of MVK-oxide and MACR-oxide^47^ are 2–10 times larger than those of CH_2_OO in the 363–394 nm region^38^. The fitting shows no other constituents than the absorption from CH_2_OO and MACR, indicating essentially zero stabilization of C_4_ CIs (<1 × 10^9 ^cm^−3^) in our condition, and all sCIs are essentially stabilized CH_2_OO at the low pressure (7.5 Torr).

**Fig. 3:**
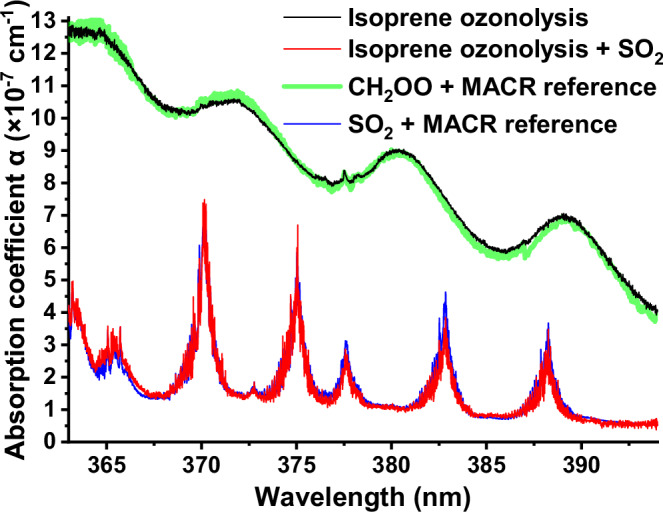
UV spectra of isoprene ozonolysis with the presence (red) and absence (black) of SO_2_ scavenger, and comparison with the fitted spectra of SO_2_ + MACR (blue) and CH_2_OO + MACR (green) from the reference spectra^38,45,52,64,65^. Source data are provided as a Source Data file.

Subsequently, we added an excess amount of SO_2_ (9 × 10^14 ^cm^−3^), which can effectively react with sCIs^17,41,48,49^ with a large rate constant (such as 3.9 × 10^−11^ cm^3^ s^−1^ with CH_2_OO^19,38^), as a scavenger to confirm that the spectral features were from CIs. Figure 3 shows the spectra of isoprene ozonolysis without and with SO_2_, in comparison with the summed reference spectra of CH_2_OO (1.2 × 10^11 ^cm^−3^) and MACR (2.6 × 10^12 ^cm^−3^), and SO_2_ (9 × 10^14 ^cm^−3^) and MACR (2.6 × 10^12 ^cm^−3^). The broad spectral features of CH_2_OO in isoprene ozonolysis between 363–394 nm vanished after SO_2_ was added to the system, replaced by the sharp peaks of SO_2_. The fitting shows that the CH_2_OO concentration decreased by more than 95%. Thus, chemical titration strongly indicates the successful measurement of CIs in isoprene ozonolysis.

The concentration profile of CH_2_OO along the flow cell, calculated from kinetic simulations of the isoprene ozonolysis reaction network (the final, optimized version is listed in Supplementary Table 1), is plotted in Supplementary Fig. 4, which indicates that shorter residence time favors higher concentration of sCIs. As the residence time increases, byproducts such as carbonyls and organic acids build up, which react rapidly with sCIs^38^. Thus, residence times less than 1 s were used to reduce the consumption of CH_2_OO to ensure that its concentration was high enough for the following direct kinetic studies. To remove the potential absorption contributions of byproducts (such as carbonyls or any broad absorptions), difference spectra (net absorption spectra of sCIs) were obtained for the kinetic measurements of CH_2_OO concentrations, especially at longer residence time, as shown in Supplementary Figs. 5–7^50^.

**Fig. 4:**
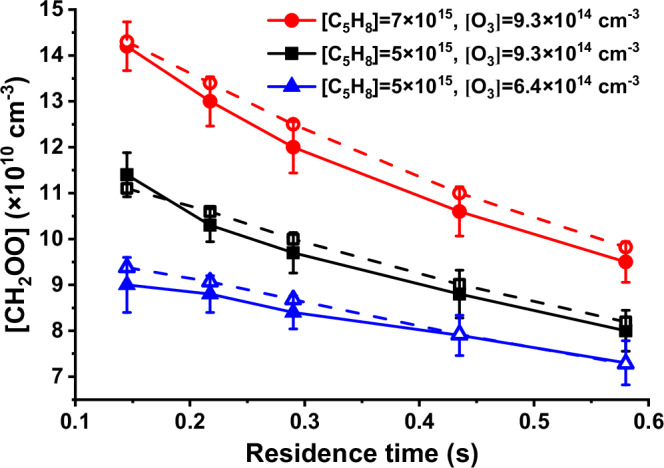
Concentration time profile of CH_2_OO in isoprene ozonolysis at different residence time under varied reaction conditions at 7.5 Torr and 293 K. Solid symbols: experimental data; open symbols: kinetic simulation; red circles: [isoprene] = 7 × 10^15 ^cm^−3^ and [O_3_] = 9.3 × 10^14 ^cm^−3^; black squares: [isoprene] = 5 × 10^15 ^cm^−3^ and [O_3_] = 9.3 × 10^14 ^cm^−3^; blue triangles: [isoprene] = 5 × 10^15 ^cm^−3^ and [O_3_] = 6.4 × 10^14^ cm^−3^. Error bars of [CH_2_OO] are estimated by propagating spectral baseline fluctuations (Δα) using Eq. (1), where Δα is the full baseline excursion across 900 spectral data points in the 378–389 nm region. [CH_2_OO] are determined from difference spectra between ozonolysis and ozonolysis + SO_2_ conditions (as detailed in Supplementary Fig. 5-7), which remove potential UV absorption contributions from carbonyls, O_3_, and other broad background. Source data are provided as a Source Data file.

In the CH_2_OO kinetic study shown in Fig. 4, the residence time was varied between 0.15 and 0.6 s while the initial concentrations of isoprene and ozone were changed in the ranges of 5–7 × 10^15^ and 6.4–9.3 × 10^14 ^cm^−3^, respectively. Concentration time profiles of CH_2_OO from experiments are compared to kinetic simulations to constrain the model and determine the important parameters. Alongside CH_2_OO, concentrations of other species with absorption features in the near-UV region, including HCHO^51^ and MACR^52^, were also monitored and compared to the kinetic simulation to achieve more accurate results, as shown in Supplementary Fig. 8. The stabilized CH_2_OO yield was determined to be 21 (±1) %. The rate coefficients of the reaction of CH_2_OO with isoprene, O_3,_ and HCHO reaction, major CI reaction pathways under our experimental condition, were determined from the fit to be 1.5 (±0.2) × 10^–15^, 4.5 (±0.4) × 10^–14^, and 3.0 (±0.3) × 10^–12^ cm^3^ s^−1^, respectively. The uncertainties of measuring the stabilization yield and rate coefficients were estimated based on the 1σ standard deviations when all the rate constants in the kinetic model and the sCI yield were varied randomly with a Gaussian distribution within ±10% (relative value), as shown in Supplementary Fig. 9^38^.

The sCI yield was further confirmed by performing sCI scavenging experiments with SO_2_ and calculated from the ratio of the consumed SO_2_ to the consumed O_3_. Details of titration experiments are the same as our previously reported sCI measurement experiments^48,49^. Representative scavenging spectra of isoprene ozonolysis in the 323.5–325.5 nm region are shown in Supplementary Fig. 10a. The pressure was varied at 5–13 Torr, and the total sCI yields were determined to be close to 22%, as shown in Supplementary Fig. 10b, which not only agrees well with the direct detection experiment, but also confirms that little to no C_4_ CIs are produced cold in the low-pressure region.

## Discussion

In this work, the use of a flow reactor under laboratory conditions enables the investigation of both primary and secondary reactions in isoprene ozonolysis, providing an intermediate link between measurements of individual rate constants and environmental chamber-scale mechanistic studies, and thereby ultimately leading to improved atmospheric chemistry modeling. The measurements of sCIs in this study were performed at 5–13 Torr but could be extended to higher pressure when a higher pumping speed is available to maintain the short residence time.

Notably, the yield of stabilized CH_2_OO was found to be 21% at 7.5 Torr. At 5–13 Torr, we find that essentially all nascent sCIs are CH_2_OO, with a stabilized yield of ~21% and little to no stabilization of C_4_ CIs. In comparison, at 760 Torr, previous studies give a consistent total sCI yield in isoprene ozonolysis of 61 (±9) %^17,41,53^ (as summarized in Supplementary Table 2). By directly detecting CH_2_OO in isoprene ozonolysis and quantifying its nascent stabilization at low pressure, this work provides the types and yields of nascent sCIs in the net isoprene + ozone reaction (prior to any pressure stabilization of CIs) and a boundary condition for pressure-falloff parameterizations^54^. Although some previous studies reported negligible amount of stabilized C_4_ CIs at ambient pressure^17,55^, recent pressure-dependence analyses by Hakala et al.^54^ showed that a portion of CH_2_OO is born stable (as directly confirmed in this study), with modestly additional pressure stabilization (stabilized CH_2_OO yield increases from ~20% to ~30% when pressure increases from zero to 760 Torr), whereas most of the pressure sensitivity arises from stabilization of MACR-oxide and MVK-oxide (stabilized C_4_ CI yield increases from zero to ~30% when pressure increases from zero to 760 Torr). Previous works by Neeb et al.^56^ also showed that stabilized CH_2_OO yield is ~30% at 760 Torr^54,56,57^. Together with our results of ~21% stabilized yield of CH_2_OO and ~ 0% stabilized yield of C_4_ CIs at low pressure and the total sCI yield of ~61% at atmospheric pressure reported previously^17,41,53^, these literature results imply that a substantial fraction of sCIs under atmospheric conditions may arise from larger C_4_ CIs (MVK-oxide and MACR-oxide). A recent atmospheric-pressure study further reported ~11% C_4_ sCIs formed in isoprene ozonolysis^58^, supporting the presence of non-negligible C_4_ sCIs contributions. In summary, this study shows that at low pressure, there are 21% stabilized CH_2_OO and no stabilized C_4_ CIs (from direct experimental measurements of CIs), while the remaining CIs are estimated to consist of 37% hot CH_2_OO and 42% hot C_4_ CIs (based on kinetic simulations and experimental constraints of HCHO, O_3_, and MACR, as detailed in Supplementary Table 1 and Methods). At 760 Torr, previous studies indicate that the total sCI yield increases to ~61%, reflecting additional stabilization of both CH_2_OO and C_4_ CIs due to collisions.

Theoretical work by Zhang et al.^57^ demonstrated that collisional stabilization strongly depends on the size of the CIs and pressure, indicating that a significant fraction of CH_2_OO is born cold, because excess energy from primary ozonide decomposition is largely partitioned into the larger coproduct MVK or MACR; while the larger C_4_ CIs (MACR-oxide and MVK-oxide) are born comparatively hot with the co-fragment HCHO taking away less energy^37^. The stabilization fraction of C_4_ CIs was theoretically predicted to increase rapidly with increasing pressure, from only 4% at 7.5 Torr to ~54% at 760 Torr, underscoring their strong pressure dependence^57^. In the atmosphere, because CH_2_OO reacts efficiently with water, previous chamber studies infer that sCIs are unlikely to contribute substantially to gas-phase SO_2_ oxidation under typical boundary-layer conditions^17^. However, a recent study by Lin et al.^59^ indicates that not all the reactions of isoprene-derived CIs are fast and that the lifetime of anti-MACR-oxides is much longer than previously considered. Caravan et al.^16,60^ compared the lifetime and reaction pathways of C_4_ sCIs and CH_2_OO in the troposphere, showing that CH_2_OO is efficiently removed by water dimer, whereas C_4_ sCIs are weakly quenched by water but react rapidly with SO_2_ and organic acids, with global modeling indicating that the MVK-oxide + SO_2_ reaction can contribute to sulfate aerosol formation. Their observations, in combination with our sCI yield measurements and the discussion above, further suggest that C_4_ sCIs could play important roles in tropospheric oxidation chemistry^16,17,41,60–62^. Global simulations suggest that the sCI chemistry may exert a broader influence on tropospheric composition than currently captured^43^. The experimental benchmarks established in this study provide direct constraints on the sCI formation and kinetics in isoprene ozonolysis, representing a critical advance for atmospheric modeling frameworks.

## Methods

As shown in Supplementary Fig. 2, a cylindrical flow cell (length 57 cm, diameter 2.54 cm) was used as a fast reactor for ozonolysis reactions. The isoprene liquid sample (Thermo Scientific, 98%) was mixed with the N_2_ dilution gas and flowed into the reactor together with O_3_ (~3–5% in O_2_) produced by an ozone generator (ENALY model 1000BT-12). SO_2_ (~4% in N_2_) was mixed with alkene before O_3_ when sCIs need to be scavenged for confirmation of sCI identity or sCI yield measurements. Reactant flows were regulated by mass flow controllers (MFCs, Aalborg model GFC17S-EAL6-A0), providing stable and continuous flow rates. The total pressure inside the flow reactor was monitored using a Cole-Parmer pressure gauge (model EW-68936-00). An inline valve at the reactor outlet was used to adjust the effective pumping speed of the vacuum pump (Welch model 1397), thereby allowing constant pressure across different flow rates. The flow parameters of the reactor are listed in Supplementary Table 3, which shows that the radial diffusion in the flow cell can be ignored under experimental conditions, and the flow reactor can be reasonably seen as a plug flow reactor (PFR).

The average concentration of the targeted species was measured using CRDS according to the following equation:

1 $$\alpha={\sum }_{i}{\sigma }_{i}\left(\lambda \right){N}_{i}+f\left(\lambda \right)=\frac{{{{\rm{L}}}}}{{{{{\rm{cl}}}}}_{{{{\rm{s}}}}}}\left(\frac{1}{\tau }-\frac{1}{{\tau }_{0}}\right)$$

where $\alpha$ is the absorption coefficient, ${N}_{i}$ is the number density (molecules per unit volume) of the *i*-th absorber, ${\sigma }_{i}$ is the absorption cross-section of the *i-*th species at wavelength $\lambda$, ${{{\rm{c}}}}$ is the speed of light, ${{{\rm{L}}}}$ is the distance between the two mirrors, ${{{{\rm{l}}}}}_{{{{\rm{s}}}}}$ is the sample length (distance between the sample input and output), $\tau$ is the ring-down time when absorber species are in the cavity, ${\tau }_{0}$ is the ring-down time in an empty cavity, parameter $f\left(\lambda \right)$ accounts for the unidentified broad extinction contribution at varying wavelengths. A Surelite Continuum 532 nm Nd: YAG laser pumped a Lambda-Physik dye laser to generate 10 Hz pulse outputs at 640–650 nm, 670–690 nm, and 720–790 nm (using DCM dye in methanol, Pyridine 1 in MeOH, and Styryl 8 in MeOH and DMSO, respectively). Inrad Autotracker Ⅲ produced second harmonics in the range of 320–325 nm, 335–345 nm, and 360–395 nm. Three pairs of highly reflective mirrors (>99.99%, Layertec GmbH) centered at 330 nm, 360 nm, and 395 nm were used to obtain a baseline ringdown time ${\tau }_{0}$ from 5 to 16 μs. With a long effective path and high sensitivity ($\alpha$_min_ ~ 1 × 10^-8 ^cm^−1^), CRDS can capture signals from transient species of low concentrations, allowing quantification of CH_2_OO concentration with a sensitivity of Δ[CH_2_OO] = 5.0 × 10^9 ^cm^−3^.

The rate coefficient of isoprene + O_3_ is relatively slow (1.30 × 10^−17 ^cm^3 ^s^−1^) compared to the fast depletion reactions of stabilized CH_2_OO, resulting in the low concentration of stabilized CH_2_OO. Under typical experimental conditions, the concentration of CH_2_OO can achieve ~1–10 × 10^10 ^cm^−3^, which was high enough to be measured by near-UV CRDS. To accurately model the chemical kinetics occurring inside the PFR, a mechanism of isoprene ozonolysis was constructed mainly based on the branching ratios in isoprene work of Nguyen et al.^17^ and our previous CH_2_OO kinetic study in ethene ozonolysis^38^, and expanded to include more CI reactions and other secondary reactions with kinetic data reported previously. The reaction network was later finalized by the optimized kinetic parameters and sCI yields in this work, as shown in Supplementary Table 1^14,15^. The simulation was performed with the chemical kinetic software package KINTECUS^63^. Simulation parameters involved in the CI production and consumption reactions in Supplementary Table 1 (such as yields, branching ratios, and rate constants) were obtained by fitting to the measured CH_2_OO, HCHO, and MACR concentration time profiles^38,45,52,64,65^. The total CH_2_OO yield, which is complementary to the primary HCHO yield, was constrained by combining time-resolved measurements of HCHO and O_3_ with kinetic simulations, in which secondary HCHO formation pathways were explicitly accounted for (see Supplementary Table 1). MACR measurements provide an additional constraint on product branching. Branching ratios of all the primary pathways of isoprene ozonolysis were established (see Supplementary Table 1). The PFR was modeled as a series of continuous stirred tank reactors (CSTR), with the output of each CSTR serving as the input of the next along the flow path^38^.

## Supplementary information

Supplementary Information

Transparent Peer Review file

## Source data

Source data

## Data Availability

Source data are provided with this paper.
