# Supplementary material for: Direct measurement of Criegee intermediates in isoprene ozonolysis
Source: Nat Commun. 2026 May 20;17:6635. doi: 10.1038/s41467-026-73307-6 (PMC13381745; doi:10.1038/s41467-026-73307-6)
Supplement: Supplementary file 1 — Supplementary Information [file 41467_2026_73307_MOESM1_ESM.pdf]

Supplementary information for

Direct measurement of Criegee intermediates in isoprene ozonolysis

Authors: Lei Yang[1], Katia Hatem[1], Mixtli Campos-Pineda[1,3], and Jingsong Zhang[1,2*]

Affiliations:

[1]Department of Chemistry, University of California, Riverside, 92521, USA.

[2]Air Pollution Research Center, University of California, Riverside, 92521, USA.

[3]Present address: Centre for Research into Atmospheric Chemistry, University College Cork, Cork, Ireland.

[*]Corresponding author. Email: jingsong.zhang@ucr.edu

Contents:

Supplementary Figs. 1 to 10
Supplementary Tables 1 to 3
Supplementary references

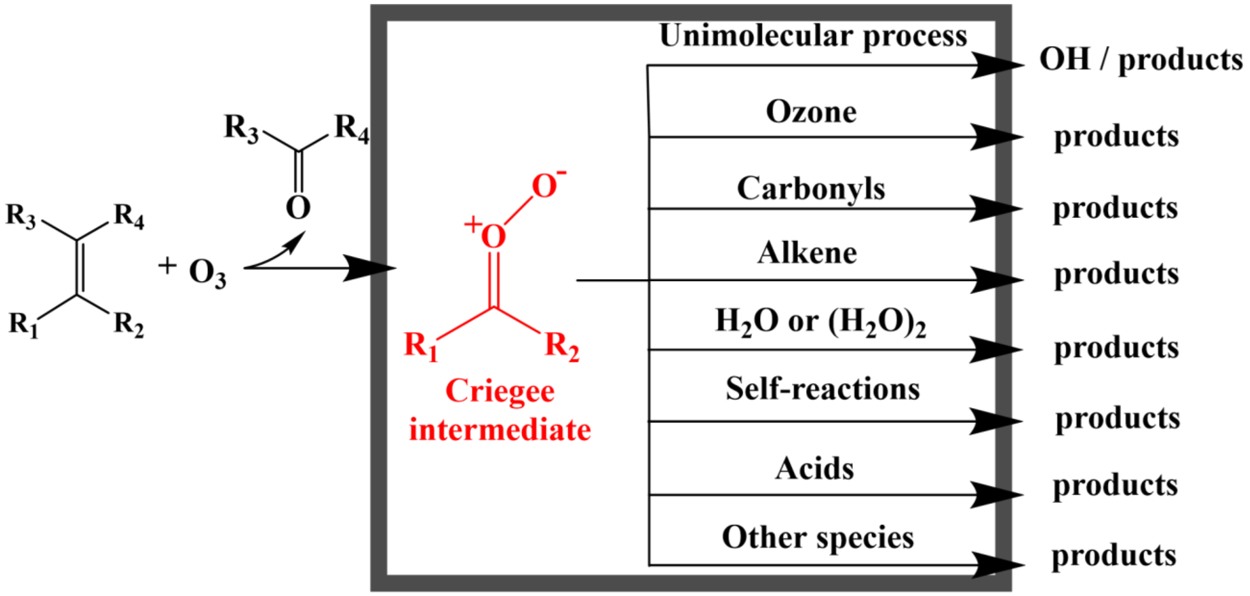

Supplementary Fig. 1. Measuring the end products can only provide indirect information on CI reaction mechanisms in ozonolysis and needs many assumptions in the black box.

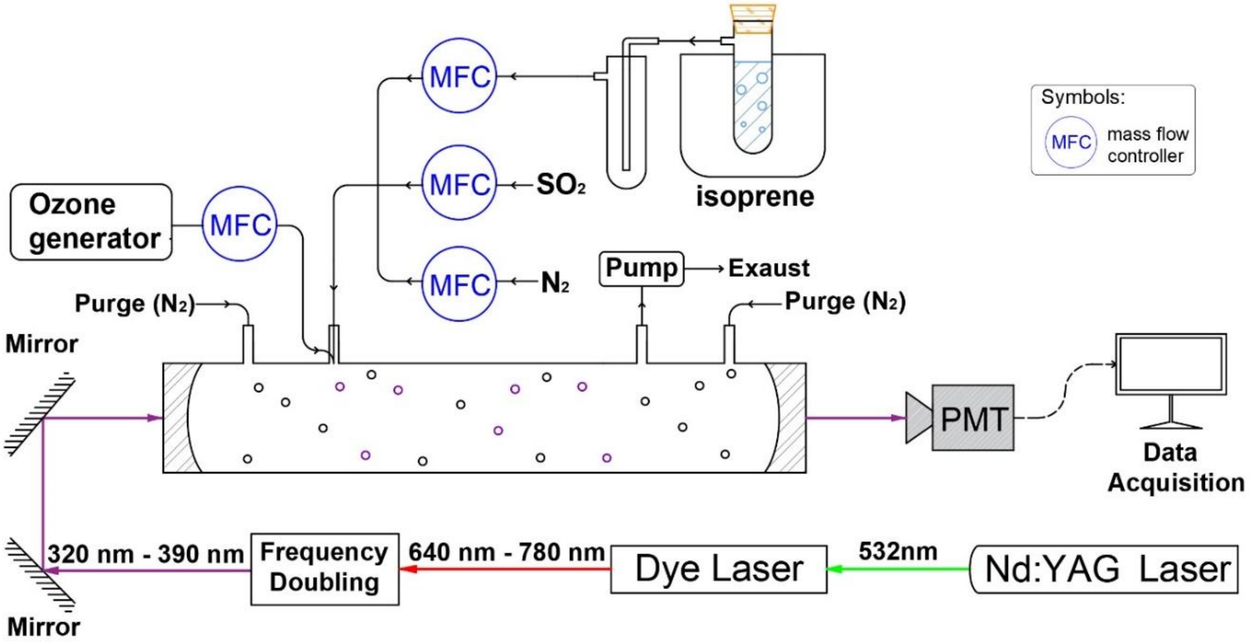

Supplementary Fig. 2. Schematic of the flow reactor used for isoprene ozonolysis experiments. Isoprene was mixed with nitrogen as a buffer gas (SO_2 added only for scavenging experiments). The isoprene mixture reacted with ozone in oxygen from an ozone generator from the reactor inlet throughout the reactor cell. The reactor was also used as a cavity for the CRDS measurements. Mass flow controllers are labelled as MFC.

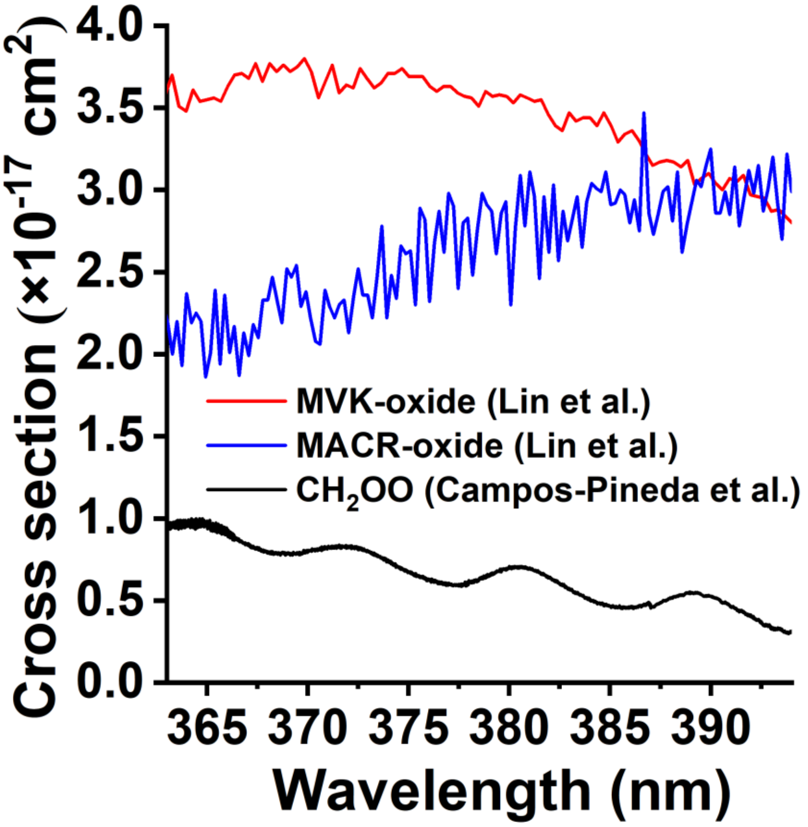

Supplementary Fig. 3. Absorption cross sections of CIs from literature, including CH_2OO (Campos-Pineda et al.[1]), MVK-oxide and MACR-oxide (Lin et al.[2]). The fitting in Fig. 2 shows that the concentrations of MVK-oxide and MACR-oxides were orders of magnitude smaller than that of CH_2OO, indicating near-zero stabilization of C_4 CIs in our conditions.

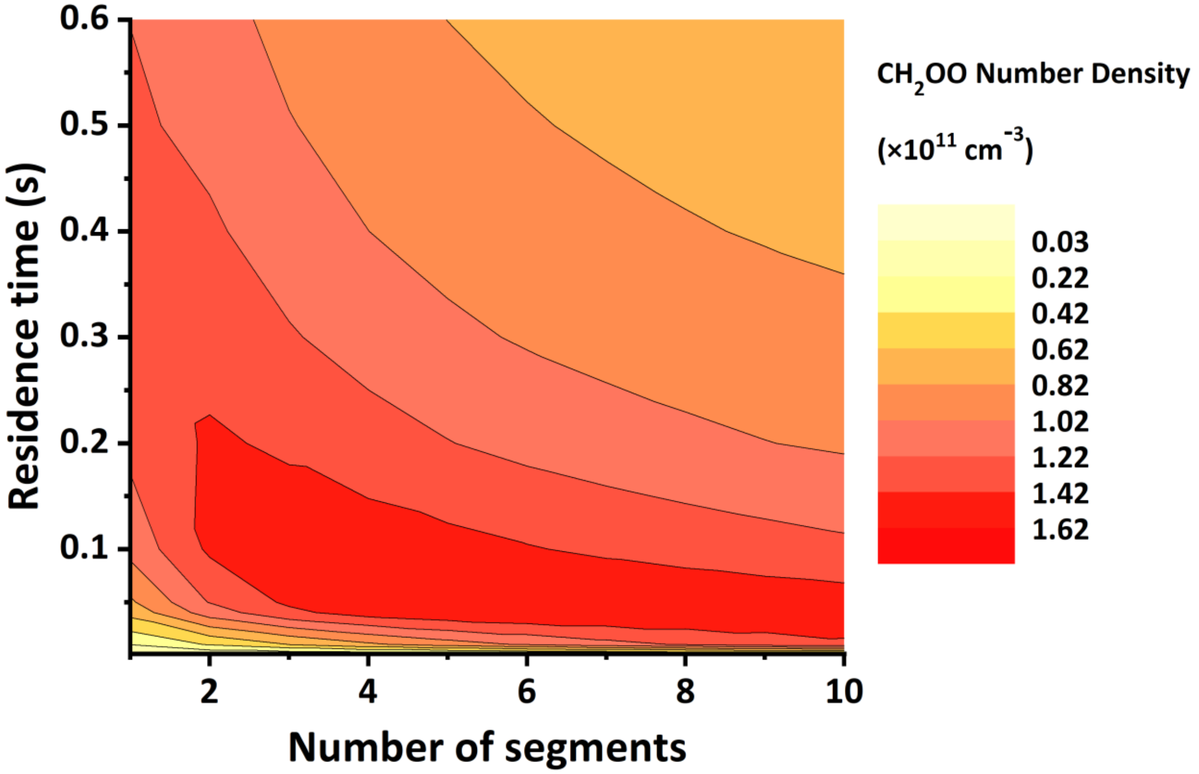

Supplementary Fig. 4. Concentration profile of CH_2OO along the reactor of isoprene ozonolysis at different residence times. The segments of the flow cell were modelled as CSTRs, and the whole reactor was modelled as CSTRs in tandem[1].

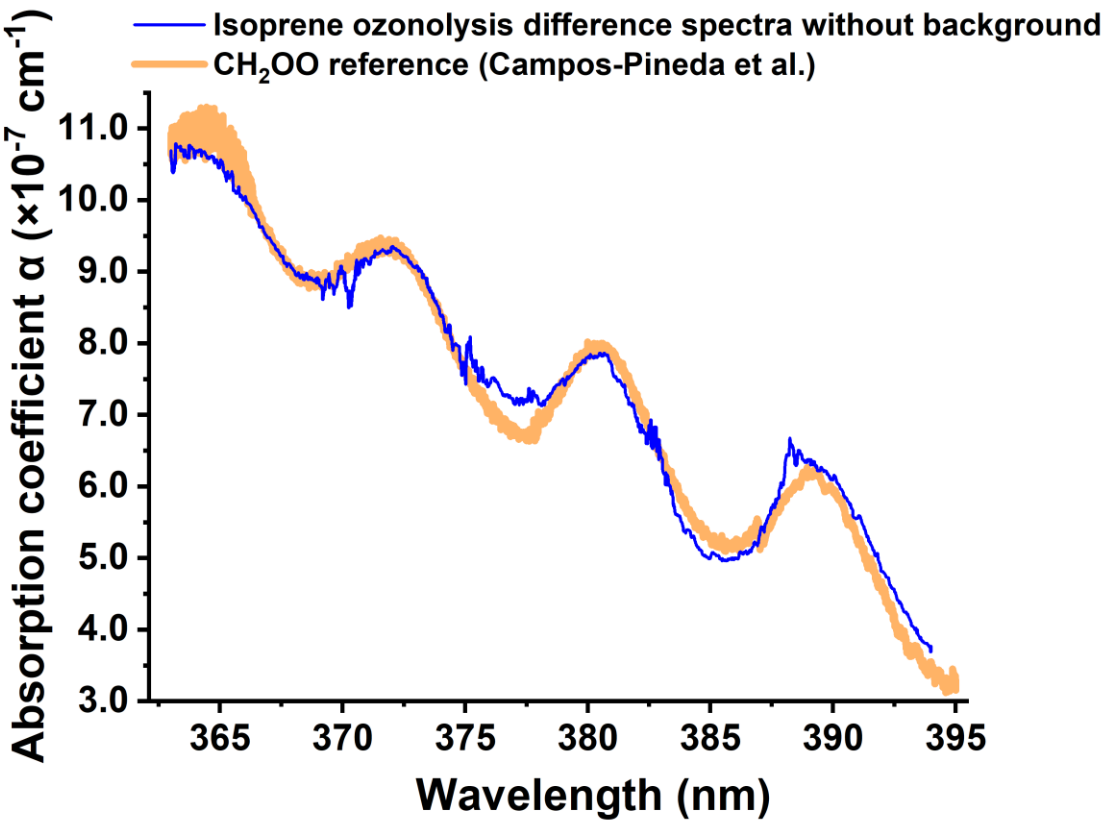

Supplementary Fig. 5. Representative “difference spectra” (blue) of isoprene ozonolysis after removing the broad absorption background from byproducts (such as carbonyls) other than CIs (the net absorption spectra of sCIs), compared to the CH_2OO reference (orange) scaled from Campos-Pineda et al.[3]. The absorption background of byproducts can be calculated from spectra of “isoprene ozonolysis + SO_2” minus the SO_2 reference. The difference spectra are obtained from Fig. 3 and essentially equal to the “isoprene ozonolysis spectra” plus “SO_2 reference” minus the “isoprene ozonolysis + SO_2 spectra”. The difference spectra in blue have some residual noise due to imperfect removal of the SO_2 features.

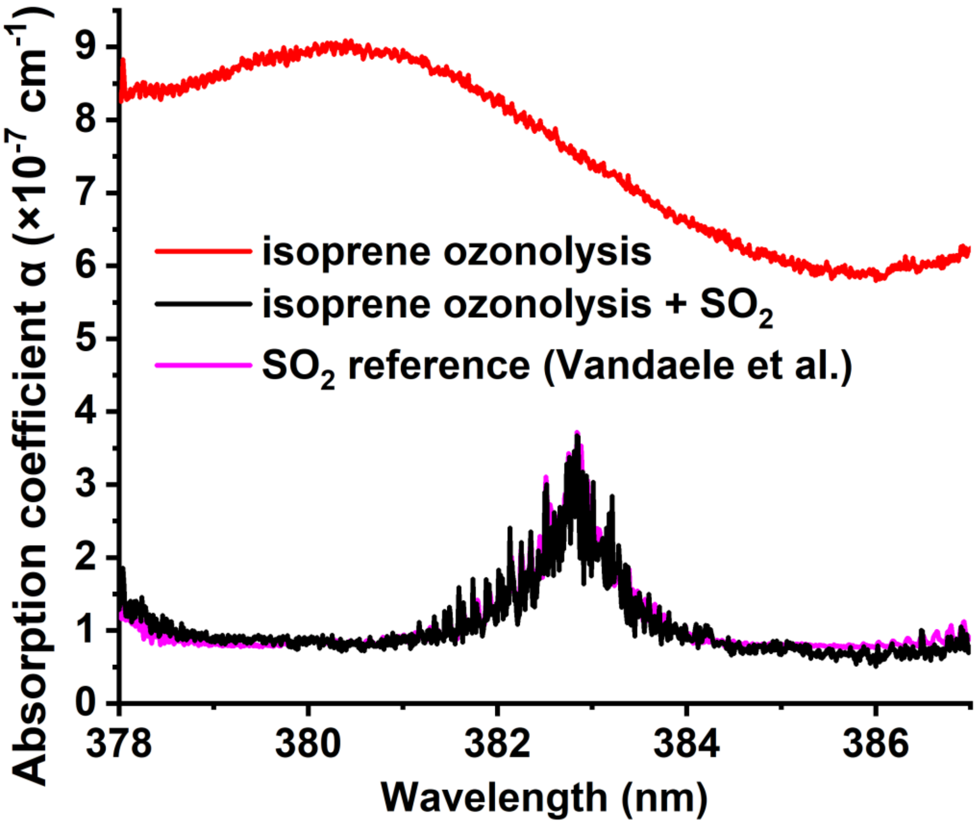

Supplementary Fig. 6. Representative original spectra for kinetic study in Fig. 4. The difference spectra (the net absorption spectra of sCIs) shown in Supplementary Fig. 7 are from the red (isoprene ozonolysis) + pink (SO_2 reference) – black (isoprene ozonolysis + SO_2) curve, which may introduce some noise around the 382.5-nm SO_2 peak, but would remove the contributions from potential byproducts (as detailed in Supplementary Fig. 5).

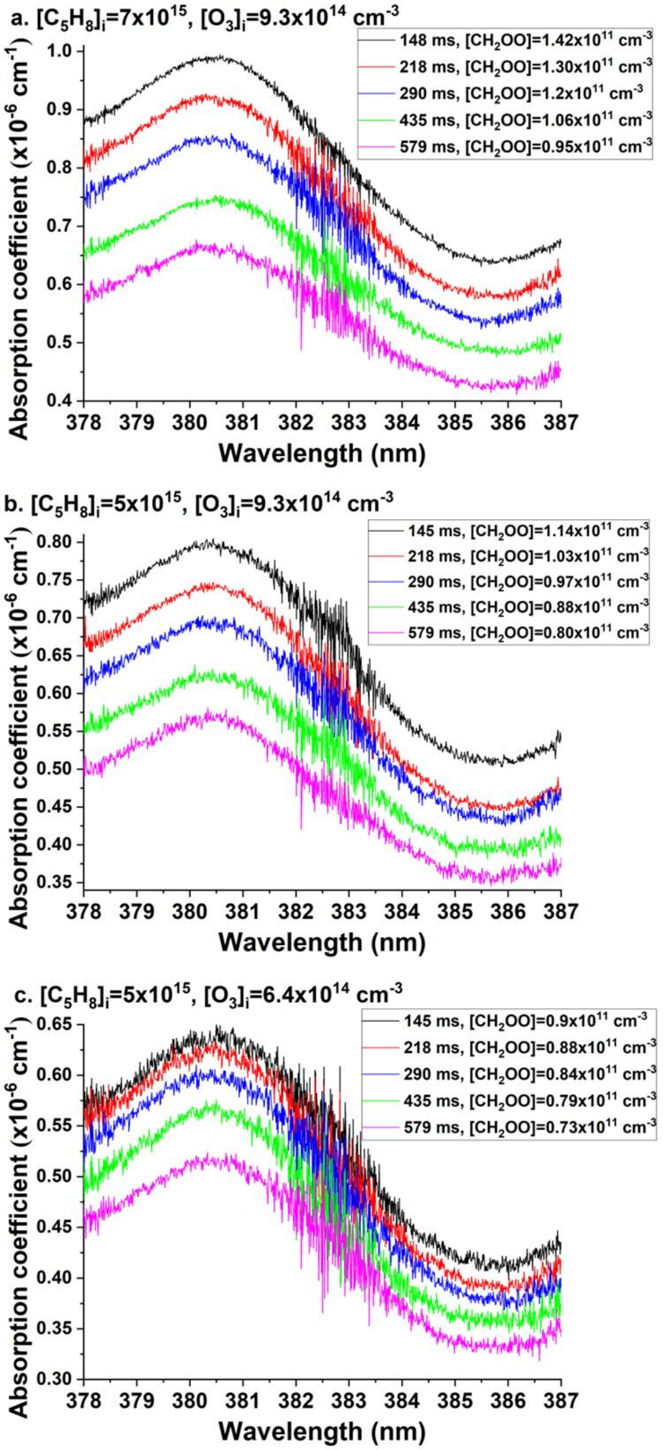

Supplementary Fig. 7. Difference absorption spectra from the isoprene ozonolysis reactions without and with the SO_2 scavenger (the net absorption spectra of CH_2OO, used to obtain the CH_2OO concentrations in Supplementary Fig. 4), under different residence times with the varied initial isoprene and ozone concentrations of **(a)** 7×10^{15} and 9.3×10^{14} cm^{-3}, **(b)** 5×10^{15} and 9.3×10^{14} cm^{-3}, and **(c)** 5×10^{15} and 6.4×10^{14} cm^{-3}, respectively. Noises around 382.5 nm are due to the removal of SO_2 absorption features, as explained in Supplementary Fig. 6.

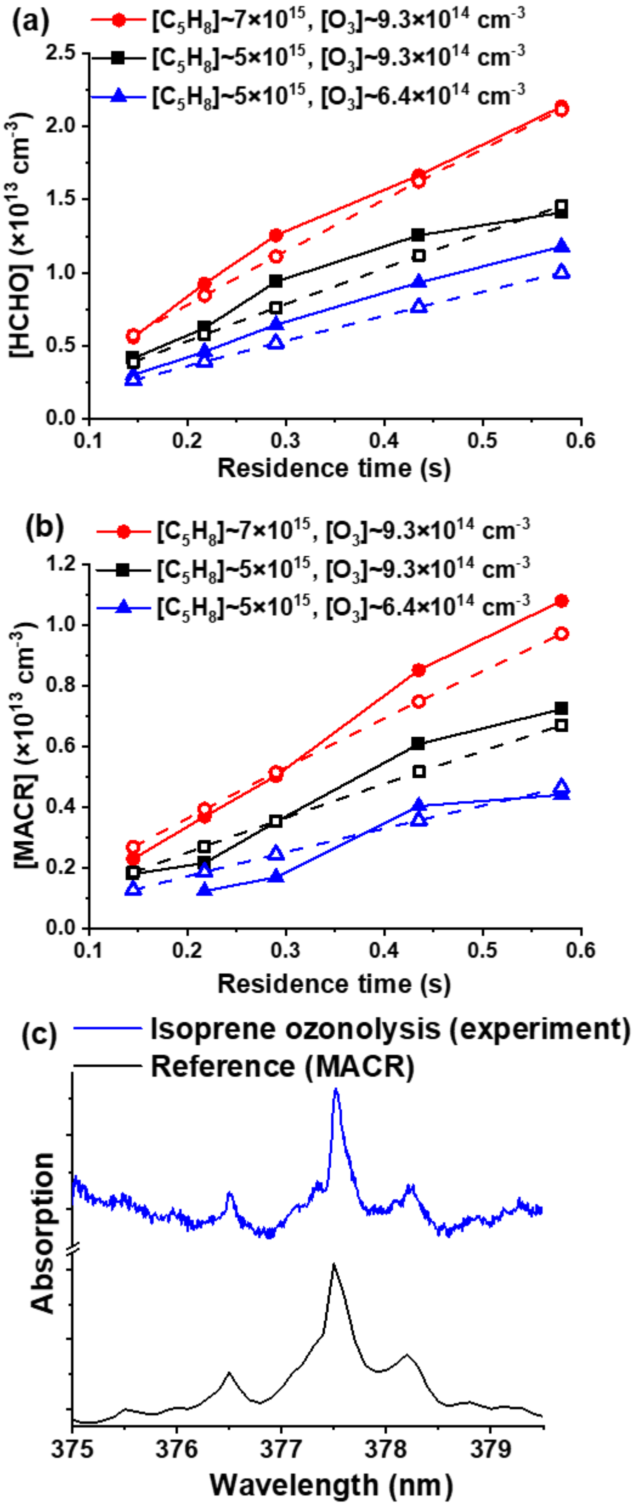

Supplementary Fig. 8. Concentration time profiles of **(a)** HCHO and **(b)** MACR in isoprene ozonolysis at different residence times under different reaction conditions at 7.5 Torr and 293 K (solid symbols: experimental data; open symbols: kinetic simulation), and **(c)** representative absorption spectra of MACR[4]. The HCHO reference spectra used are from Smith et al.[5].

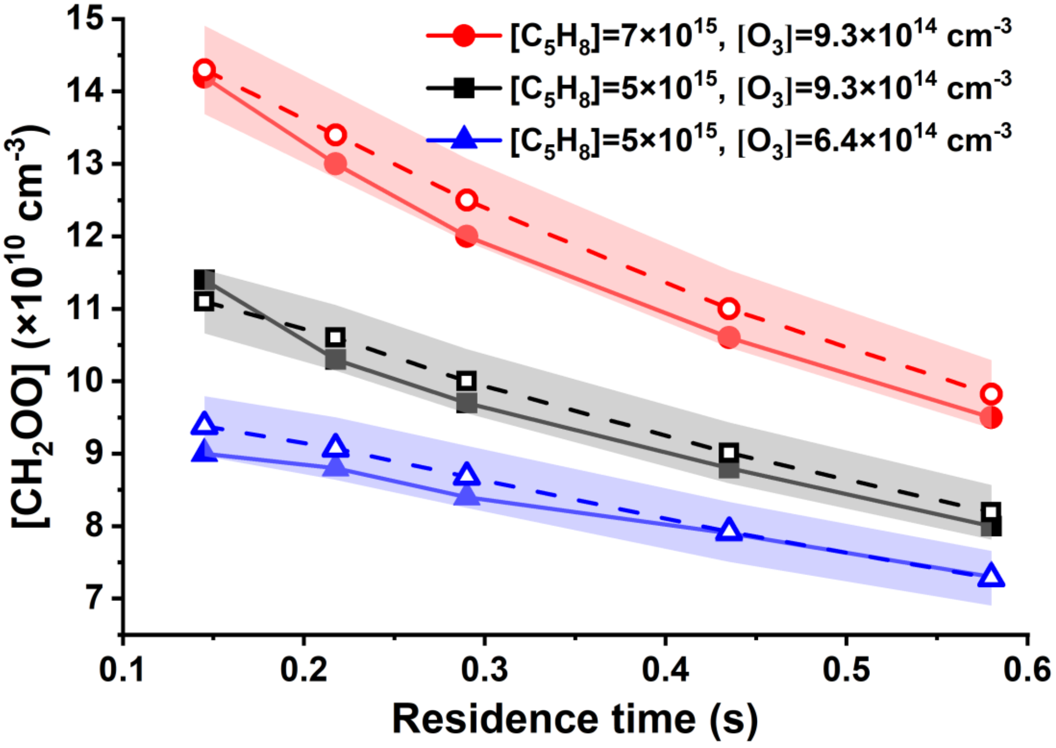

Supplementary Fig. 9. Uncertainty analysis on the kinetic parameters under different residence times at 7.5 Torr and 293 K (solid symbols: experimental data; open symbols: kinetic simulation). The error bars of the kinetic simulations (colored shades) represent 1σ standard deviations obtained from 100 simulations in which all the rate constants in the kinetic model and the sCI yield were varied randomly with a Gaussian distribution within ± 10% (relative value) and 21 (± 1) %, respectively.

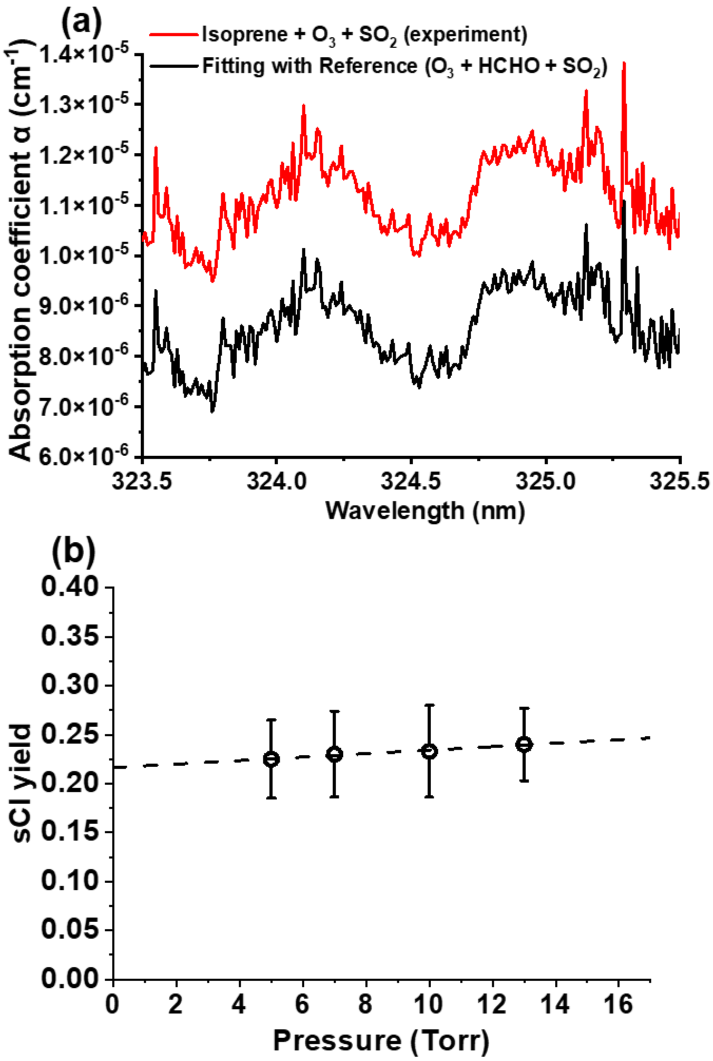

Supplementary Fig. 10. (a) Titration spectra of isoprene ozonolysis with the addition of SO_2 in the 323.5 – 325.5 nm region. The black fitting curve is offset to present the comparison with the experimental data (red). **(b)** sCI yields of isoprene ozonolysis at low pressure measured from the SO_2 titration experiments. Error bars represent one standard deviation of 3 repeated measurements.

Supplementary Table 1. Mechanism of ozonolysis of isoprene used to model concentrations of various species along the reactor. Modelling was done by dividing the PFR into CSTRs in tandem. Rate coefficients and yields are from Campos-Pineda et al.[1], Nguyen et al.[6], or IUPAC[7] unless specified in comments. The units of rate coefficients k are s^{-1} and $cm^3\ s^{-1}$ for unimolecular and bimolecular reactions, respectively.

# k	Reaction	Comments
1.30×10^{-17}	$C_5H_8 + O_3 \rightarrow$<br>$0.424HCHO+0.210CH_2OO+0.366CH_2OO^*$<br>$+0.408CH_3CCH_2CHO+0.168CH_3COCHCH_2$<br>$+0.1536CH_3CCH_2CHO_2^*+0.0384CH_3CCH_2CHOO^*$<br>$+ 0.0928CH_3CO_2CHCH_2^*+0.1392CH_3COOCHCH_2^*$	hot CI: CH_2OO^*, sCI: CH_2OO,<br>MACR: CH_3CCH_2CHO,<br>MVK: $CH_3COCHCH_2$,<br>anti-MACR-oxide: $CH_3CCH_2CHO_2^*$,<br>syn-MACR-oxide: $CH_3CCH_2CHOO^*$,<br>anti-MVK-oxide: $CH_3CO_2CHCH_2^*$,<br>syn-MVK-oxide: $CH_3COOCHCH_2^*$
#	**Stabilized CH_2OO reactions**	
4.50×10^{-14}	$CH_2OO + O_3 \rightarrow HCHO + O_2 + O_2$	Major consumption reactions of CH_2OO. Rate constants from fitting to experimental data of this work (Fig. 4)
1.50×10^{-15}	$CH_2OO + C_5H_8 \rightarrow HCHO + C_5H_8O$	
1.20×10^{-12}	$CH_2OO + HCHO \rightarrow HCOOH+HCHO$	
1.60×10^{-12}	$CH_2OO + HCHO \rightarrow CO+H_2O+HCHO$	
1.00×10^{-13}	$CH_2OO + HCHO \rightarrow HCO+OH+HCO+H$	
1.00×10^{-13}	$CH_2OO + HCHO \rightarrow CH_3CHO+ O_2$	
1.00×10^{-10}	$CH_2OO + HCOOH \rightarrow HCOOOCH+H_2O$	
5.00×10^{-13}	$CH_2OO + CH_3COCHCH_2 \rightarrow HCOOH+ CH_3COCHCH_2$	MVK, Escola et al, 2018[8]
4.40×10^{-13}	$CH_2OO + CH_3CCH_2CHO \rightarrow HCOOH+CH_3CCH_2CHO$	MACR, Escola et al, 2018[8]
0.1	$CH_2OO \rightarrow 0.7HCHO_2+0.3HCOOH$	
0.1	$CH_2OO \rightarrow HHOOC$	Wall dummy
2.41×10^{-16}	$CH_2OO + H_2O \rightarrow CH_4O_3$	Water dummy
7.40×10^{-11}	$CH_2OO + CH_2OO \rightarrow 2HCHO+O_2$	Chhantyal-Pun et al., 2015[9]
9.50×10^{-13}	$CH_2OO + CH_3CHO \rightarrow HCHO+ CH_3COOH$	Taatjes et al., 2012[10]
1.00×10^{-10}	$CH_2OO + CH_3COOH \rightarrow C_3H_6O_4$	Dummy product
1.00×10^{-11}	$CH_2OO + HCOOOCH \rightarrow H_4C_3O_5$	Dummy product
#	**Hot CH_2OO^* reactions**	
8.00×10^3	$CH_2OO^* \rightarrow CO_2+H_2$	Copeland et al., 2011[11]
2.00×10^4	$CH_2OO^* \rightarrow CO+H_2O$	
1.20×10^3	$CH_2OO^* \rightarrow H+HCO_2$	
7.00×10^2	$CH_2OO^* \rightarrow 0.9HCOOH+0.1HCO+0.1OH$	
3.20×10^4	$HCO_2 \rightarrow H+CO_2$	
#	**Hot C_4 CI unimolecular reactions**	**Dominant at low pressure**
3.00×10^6	$H_3CCCH_2CHO_2^* \rightarrow$<br>$0.3CH_3CCH_2CHO+0.3O+0.7HCHO+0.7CH_3CCHO$	hot anti-MACR-oxide
3.00×10^6	$H_3CCCH_2CHOO^* \rightarrow HCO+ CH_3CHCHO$	hot syn-MACR-oxide
3.20×10^6	$H_3CCO_2CHCH_2^* \rightarrow CH_3CO + CH_2CHO$	hot anti-MVK-oxide
2.30×10^6	$CH_3COOCHCH_2^* \rightarrow 0.3\ CH_2COCHCH_2 + 0.3\ OH +$<br>$0.7\ CH_2CHCO + 0.7\ CH_2OH$	hot syn-MVK-oxide
#	**Stabilized C_4 CI unimolecular reactions**	**Only possible at high pressure**
10	$CH_3CCH_2CHO_2 \rightarrow$<br>$0.3CH_3CCH_2CHO+0.3O+0.7HCHO+0.7CH_3CCHO$	anti-MACR-oxide (Vansco et al., 2019)[12]
2500	$CH_3CCH_2CHOO \rightarrow HCO+CH_3CHCHO$	syn-MACR-oxide (Vansco et al., 2019)[12]
2140	$CH_3CO_2CHCH_2 \rightarrow CH_3CO+CH_2CHO$	anti-MVK-oxide

		(Barber et al. 2018)[13]
33	$CH_3COOCHCH_2 \rightarrow 0.3\ CH_2COCHCH_2 + 0.3\ OH + 0.7\ CH_2CHCO + 0.7\ CH_2OH$	syn-MVK-oxide (Barber et al. 2018)[13]
4.90×10^{-3}	$CH_2CHCO \rightarrow CO + C_2H_3$	
#	**MVK/MACR consumption reactions**	
4.77×10^{-18}	$CH_3COCHCH_2+O_3\rightarrow 0.9CH_3COCHO+0.9CH_2OO^*+0.1HCHO+0.1CH_3COCHOO$	
1.10×10^{-18}	$CH_3CCH_2CHO+O_3\rightarrow 0.8CH_3COCHO+0.8CH_2OO^*+0.2HCHO+0.2CH_3COOCHO$	
1.73×10^{-11}	$CH_3COCHCH_2+OH \rightarrow CH_3COCHCH_2OH$	
3.23×10^{-11}	$CH_3CCH_2CHO+OH \rightarrow CH_3CCH_2OHCHO$	
#	**Secondary O_3**	
2.66×10^{-11}	$H+ O_3\rightarrow OH+ O_2$	
7.30×10^{-14}	$OH+ O_3\rightarrow HO_2+ O_2$	
2.00×10^{-15}	$HO_2+ O_3\rightarrow OH+2O_2$	
8.00×10^{-15}	$O+ O_3\rightarrow 2O_2$	
#	**Secondary isoprene**	
3.50×10^{-11}	$C_5H_8+O\rightarrow C_5H_8O$	
#	**HOx reactions (non-VOC)**	
5.60×10^{-12}	$H+HO_2\rightarrow H_2+O_2$	
7.20×10^{-11}	$H+HO_2\rightarrow 2OH$	
2.40×10^{-12}	$H+HO_2\rightarrow H_2O+O$	
3.50×10^{-11}	$O+OH\rightarrow O_2+H$	
5.80×10^{-11}	$O+HO_2\rightarrow OH+O2$	
1.70×10^{-15}	$O+H_2O_2\rightarrow OH+HO_2$	
6.70×10^{-15}	$H_2+OH\rightarrow H_2O+H$	
1.48×10^{-12}	$OH+OH\rightarrow H_2O+O$	
2.60×10^{-13}	$OH+OH\rightarrow H_2O_2$	Pressure dependent
1.10×10^{-10}	$OH+HO_2\rightarrow H_2O+O_2$	
1.70×10^{-12}	$OH+H_2O_2\rightarrow H_2O+HO_2$	
1.60×10^{-12}	$HO_2+HO_2\rightarrow H_2O_2+O_2$	Pressure dependent
#	**Secondary O_2 reactions**	
1.27×10^{-14}	$H+O_2\rightarrow HO_2$	Pressure dependent
1.80×10^{-16}	$O+O_2\rightarrow O_3$	Pressure dependent
5.20×10^{-12}	$HCO+O_2\rightarrow CO+HO_2$	
5.10×10^{-12}	$CH_3CO+O_2\rightarrow HCHO+OH+CO$	Pressure dependent
2.62×10^{-12}	$C_2H_3+O_2\rightarrow HCHO+HCO$	
9.70×10^{-12}	$CH_2OH+O_2\rightarrow HCHO+HO_2$	
#	**Secondary vinoxy (CH_2CHO) + O_2 reactions**	
1.60×10^{-14}	$CH_2CHO +O_2\rightarrow CH_2CO + HO_2$	
6.00×10^{-15}	$CH_2CHO +O_2 \rightarrow CHOCHO + OH$	
8.00×10^{-15}	$CH_2CHO +O_2 \rightarrow HCHO + CO + OH$	
6.00×10^{-14}	$CH_2CHO + O_2 \rightarrow OOCH_2CHO$	Dummy product
#	**HO_x reactions with VOCs**	
1.44×10^{-13}	$OH+CO\rightarrow H+CO_2$	
8.50×10^{-12}	$OH+HCHO\rightarrow H_2O+HCO$	
1.43×10^{-11}	$OH+CH_3CHO\rightarrow H_2O+ CH_3CO$	

7.50×10^{-13}	$OH+CH_3CHO\rightarrow H_2O+CH_2CHO$	
4.50×10^{-13}	$OH+HCOOH\rightarrow CH_3O_3$	Dummy product
7.90×10^{-14}	$HO_2+HCHO\rightarrow HOCH_2OO$	
1.50×10^{2}	$HOCH_2OO\rightarrow HO_2+HCHO$	
7.00×10^{-13}	$HOCH_2OO+HOCH_2OO\rightarrow HCOOH+CH_2OHOH+O_2$	
5.50×10^{-12}	$HOCH_2OO+HOCH_2OO\rightarrow 2HOCH_2O+O_2$	
6.00×10^{-12}	$HO_2+HOCH_2OO\rightarrow O_2+HOCH_2O_2H$	
4.00×10^{-12}	$HO_2+HOCH_2OO\rightarrow O_2+HCOOH+H_2O$	
2.00×10^{-12}	$HO_2+HOCH_2OO\rightarrow O_2+OH+HOCH_2O$	

Supplementary Table 2. sCI yields in isoprene ozonolysis

Yield of sCI	Reference	Methodology and conditions	Pressure
0.21 (±0.01) All CH_2OO	This work	Direct measurement on sCIs at 7.5 Torr + kinetic simulation + SO_2 loss at 5-13 Torr with titration	< 20 Torr
0.58 (CH_2OO=0.3)	Hakala et al. (2023)[14]	H_2SO_4 Formation at 600-900Torr + pressure dependence analysis on 0-1000 Torr	0-1000 Torr
0.61 (±0.09)	Nguyen et al. (2016)[6]	Product yields of MACR, MVK, HCHO, HCOOH, H_2O_2, HMHP, and HPMF at high/low [H_2O] or [SO_2]	760 Torr
0.56 (±0.03)	Newland et al. (2015)[15]	SO_2 loss	760 Torr
0.58 (±0.26)	Sipilä et al. (2014)[16]	H_2SO_4 Formation at high [SO_2]	760 Torr
0.30 (all CH_2OO)	Neeb et al. (1997)[17]	ΔHMHP	760 Torr
0.26	Hasson et al. (2001)[18]	ΔHMHP and ΔH_2O_2 at high/low [H_2O]	760 Torr
0.28	Rickard et al. (1999)[19]	Assuming 40% CH_2OO stabilization + ΔMVK and ΔMACR at high/low [SO_2]	760 Torr
0.53	Rickard et al. (1999)[19]	Assuming 95% CH_2OO stabilization + ΔMVK and ΔMACR at high/low [SO_2]	760 Torr
0.57	Zhang et al. (2002)[20]	Theoretical	760 Torr

Supplementary Table 3. Flow parameters of the reactor under experimental conditions. Da and Pe are the Damkoehler and Peclet numbers, respectively.

**Characteristic Time**	**Value**	**Description**	
$\mathbf{t_{ck}}$	0.1	Chemical reaction	
$\mathbf{t_{sd,R}}$	0.038	Radial species diffusion	
$\mathbf{t_{fc,R}}$	0.06	Radial forced convection	
$\mathbf{t_{fc,L}}$	3	Axial forced convection	
**Negligible Axial Diffusion**			
**Parameter**	Value	Description	Criterion*
$\mathbf{(t_{fc,R})^2/(t_{sd,R} \times t_{ck})}$	0.01	Da/Pe^2	< 0.1
$\mathbf{t_{fc,R}/ t_{sd,R}}$	1.6	Pe^{-1}	< 0.06
$\mathbf{t_{ck}/ t_{fc,L}}$	0.06	Reaction time vs. longitudinal convection	<< 1
**Negligible Poiseuille Flow**			
$\mathbf{t_{sd,R}/ t_{fc,R}}$	0.6	Pe	< 100
$\mathbf{t_{sd,R}/ t_{fc,L}}$	0.012	Radial diffusion mixing along the reactor	< 0.5
$\mathbf{t_{sd,R}/ t_{ck}}$	4×10^{-3}	Da	< 1
$\mathbf{t_{fc,R}/ t_{fc,L}}$	6×10^{-3}	Da/Pe	< 0.05

*Criteria from Cutler et al.[21] and references therein.

Supplementary references

1. Campos-Pineda, M., Yang, L. & Zhang, J. Direct measurement of the Criegee intermediate CH_2OO in ozonolysis of ethene. *Nat. Commun.* **16**, 6515 (2025).
2. Lin, Y.-H., Takahashi, K. & Lin, J. J.-M. Absolute Photodissociation Cross Sections of Thermalized Methyl Vinyl Ketone Oxide and Methacrolein Oxide. *Phys. Chem. Chem. Phys.* **24**, 10439-10450 (2022).
3. Foreman, E. S. *et al.* High Resolution Absolute Absorption Cross Sections of the $B^1A'-X^1A'$ Transition of the CH_2OO Biradical. *Phys. Chem. Chem. Phys.* **17**, 32539-32546 (2015).
4. Meller, R. Personal Communication to E.-P. Röth, R. Ruhnke, G. Moortgat, R. Meller, and W. Schneider, Berichte des Forschungszentums Jülich, jül-3341. (1990).
5. Smith, C. A., Pope, F. D., Cronin, B., Parkes, C. B. & Orr-Ewing, A. J. Absorption Cross Sections of Formaldehyde at Wavelengths from 300 to 340 nm at 294 and 245 K. *J. Phys. Chem. A* **110**, 11645-11653 (2006).
6. Nguyen, T. B. *et al.* Atmospheric Fates of Criegee Intermediates in the Ozonolysis of Isoprene. *Phys. Chem. Chem. Phys.* **18**, 10241-10254 (2016).
7. Cox, R. A. *et al.* Evaluated Kinetic and Photochemical Data for Atmospheric Chemistry: Volume VII – Criegee intermediates. *Atmos. Chem. Phys.* **20**, 13497-13519 (2020).
8. Eskola, A. J. *et al.* Direct Kinetics Study of CH_2OO + Methyl Vinyl Ketone and CH_2OO + Methacrolein Reactions and an Upper Limit Determination for CH_2OO + CO Reaction. *Phys. Chem. Chem. Phys.* **20**, 19373-19381 (2018).
9. Chhantyal-Pun, R., Davey, A., Shallcross, D. E., Percival, C. J. & Orr-Ewing, A. J. A Kinetic Study of the CH_2OO Criegee Intermediate Self-Reaction, Reaction with SO_2 and Unimolecular Reaction using Cavity Ring-Down Spectroscopy. *Phys. Chem. Chem. Phys.* **17**, 3617-3626 (2015).
10. Taatjes, C. A. *et al.* Direct Measurement of Criegee Intermediate (CH_2OO) Reactions with Acetone, Acetaldehyde, and Hexafluoroacetone. *Phys. Chem. Chem. Phys.* **14**, 10391-10400 (2012).
11. Copeland, G., Ghosh, M. V., Shallcross, D. E., Percival, C. J. & Dyke, J. M. A Study of the Ethene-Ozone Reaction with Photoelectron Spectroscopy: Measurement of Product Branching Ratios and Atmospheric Implications. *Phys. Chem. Chem. Phys.* **13**, 14839 (2011).
12. Vansco, M. F. *et al.* Synthesis, Electronic Spectroscopy, and Photochemistry of Methacrolein Oxide: a Four-Carbon Unsaturated Criegee Intermediate from Isoprene Ozonolysis. *J. Am. Chem. Soc.* **141**, 15058-15069 (2019).
13. Barber, V. P. *et al.* Four-Carbon Criegee Intermediate from Isoprene Ozonolysis: Methyl Vinyl Ketone Oxide Synthesis, Infrared Spectrum, and OH Production. *J. Am. Chem. Soc.* **140**, 10866-10880 (2018).
14. Hakala, J. & Donahue, N. M. Carbonyl Oxide Stabilization from Trans Alkene and Terpene Ozonolysis. *J. Phys. Chem. A* (2023).
15. Newland, M. J. *et al.* Atmospheric Isoprene Ozonolysis: Impacts of Stabilised Criegee Intermediate Reactions with SO_2, H_2O and Dimethyl Sulfide. *Atmos. Chem. Phys.* **15**, 9521-9536 (2015).

16. Sipilä, M. *et al.* Reactivity of Stabilized Criegee Intermediates (sCIs) from Isoprene and Monoterpene Ozonolysis toward SO_2 and Organic Acids. *Atmos. Chem. Phys.* **14**, 12143-12153 (2014).

17. Neeb, P., Sauer, F., Horie, O. & Moortgat, G. K. Formation of hydroxymethyl hydroperoxide and formic acid in alkene ozonolysis in the presence of water vapour. *Atmos. Environ.* **31**, 1417-1423 (1997).

18. Hasson, A. S., Ho, A. W., Kuwata, K. T. & Paulson, S. E. Production of Stabilized Criegee Intermediates and Peroxides in the Gas Phase Ozonolysis of Alkenes 2. Asymmetric and Biogenic Alkenes. *J. Geophys. Res. Atmos.* **106**, 34143-34153 (2001).

19. Rickard, A. R., Johnson, D., McGill, C. D. & Marston, G. OH Yields in the Gas-Phase Reactions of Ozone with Alkenes. *J. Phys. Chem. A* **103**, 7656-7664 (1999).

20. Zhang, D., Lei, W. F. & Zhang, R. Y. Mechanism of OH Formation from Ozonolysis of Isoprene: Kinetics and Product Yields. *Chem. Phys. Lett.* **358**, 171-179 (2002).

21. Cutler, A. H., Antal, M. J. & Jones, M. A Critical Evaluation of the Plug-Flow Idealization of Tubular-Flow Reactor Data. *Ind. Eng. Chem. Res.* **27**, 691-697 (1988).
