# Supplementary material for: Direct measurement of Criegee intermediates in isoprene ozonolysis
Source: Nat Commun. 2026 May 20;17:6635. doi: 10.1038/s41467-026-73307-6 (PMC13381745; doi:10.1038/s41467-026-73307-6)
Supplement: Supplementary file 2 — Transparent Peer Review file [file 41467_2026_73307_MOESM2_ESM.pdf]

nature portfolio

Peer Review File

Direct measurement of Criegee intermediates in isoprene ozonolysis

Corresponding Author: Professor Jingsong Zhang

This file contains all reviewer reports in order by version, followed by all author rebuttals in order by version.

Version 0:

Reviewer comments:

Reviewer #1

(Remarks to the Author)
This work follows the direct detection of CH2OO in ethene ozonolysis by this group last year, and now applies their technique to the atmospherically crucial reaction of ozone with isoprene. The experimental protocol is established and the measurement of CH2OO yield is rigorous and described clearly. The measurements directly quantify the CH2OO and place an upper limit on the other possible carbonyl oxide products at low pressure -- that is the extent of the new experimental data.

The implications of the work, however, rely on combination of this measurement with the body of literature data to infer that the atmospheric pressure reaction may have a larger contribution of C4 carbonyl oxides than some models have acknowledged. The role of the present measurement in establishing, rather than merely corroborating, that conclusion should be described more clearly. In addition, the appearance of a mass spectrometric determination of C4 carbonyl oxide yields from atmospheric pressure isoprene ozonolysis (ACS Earth Space Chem. 2026, 10, 2, 328–337) changes the environment for the present work (that paper probably appeared around the time this work was submitted).

From the yields implied by the product measurements (e.g., in supplemental table 2), the discrepancy to be addressed is the contrast between the CH2OO specific (like HMHP measurements) that suggest CH2OO of about 30% and the predictions of the Nguyen et al. model, which interpreted the ~60% carbonyl oxide yield as being dominated by CH2OO, based on comprehensive product characterization. However, several other chamber studies (ref. 40 and 52, for example) suggested the presence of C4 carbonyl oxides. Hakala and Donohue proposed a model that bridged these apparent discrepancies, predicting a weakly pressure dependent yield of stabilized CH2OO at ~30%, with the rest of the Nguyen et al yield attributed to C4 species. I give that background, which is in the manuscript but in a kind of confusing way, to point out that the overall picture that this work fits into had been coming together already. In that context, this measurement confirms one leg of the Hakala and Donohue proposal, of a weakly pressure-dependent stable CH2OO yield, and the Chhantyl-Pun et al. paper that recently appeared (ACS Earth Space Chem. 2026, 10, 2, 328–337), confirms the other, a significant (~11%) yield of stabilized C4 carbonyl oxides. As a result, and given that the C4 yield measurement has just appeared, I recommend that the authors reconsider their discussion section; first I am not sure how "dominant" was the belief that CH2OO is the only stabilized carbonyl oxide in atmospheric pressure ozonolysis, and second because with the other recent result this provides direct-detection confirmation of a framework that could unify the picture of carbonyl oxide formation in isoprene ozonolysis.

Reviewer #2

(Remarks to the Author)
This work claims, as far as I know correctly, to be the first direct measurement of carbonyl oxides from isoprene ozonolysis. It is a very careful experimental study in a low-pressure (7 torr) flow cell using UV CRDS spectroscopy, including a thorough kinetic analysis as well as complementary experiments with an sCI selective scavenger (SO2) to confirm the observed signals are consistent with a carbonyl oxide - specifically formaldehyde oxide, CH2OO.

The results will be broadly interesting to both the physical chemistry and atmospheric chemistry communities. Direct

observation of sCI from ozonolysis has been extremely difficult, as ozonolysis produces a rich set of products quite quickly, and the sCI concentrations are often very low (and they are very short lived). They however play an important role in the atmosphere, where atmospheric modeling (and field observations) have shown that sCI + SO2 can be a significant source of gas-phase H2SO4 and so an important contributor to atmospheric new-particle formation.

I believe that the core finding - experimental confirmation of a 20% sCI yield at 7 torr, consisting almost entirely of CH2OO - is of sufficiently high interest to merit publication in Nature Communications. It is also my opinion that the core of the manuscript as presented, along with the supplemental information, presents this case clearly.

However, I do have some reservations about the discussion and the context, which in my opinion are in some places an oversell. Specifically:

1. In my opinion there was very strong evidence that CIs form in atmospheric isoprene ozonolysis. I would have been absolutely stunned if a paper appeared claiming this was not so. Claiming this as a fundamental "unresolved" question thus strikes me as too strong an assertion. A major reason is that, not only is there strong evidence from scavenger studies (not just in environmental chambers, but also pressure-dependent flow tubes in the cited Hakala paper), but complementary results, not discussed in this paper, addressing OH yields from isoprene ozonolysis are also strongly consistent with CI production. Specifically, Kroll et al GRL 2001 described OH formation measurements using a highly accurate atmospheric laser induced fluorescence instrument, showing a modestly decreasing OH yield from about 20% at 10 torr to about 10% at 40 torr. This is consistent with OH formation largely by excited C4 CI starting to be stablized over this range.

that brings me to

2. The discussion as worded suggests that a significant role for C4 sCI approaching atmospheric pressure is a new interpretation for this manuscript. I find that surprising, as this is exactly the argument made in the Hakala paper, which concludes that about half of the sCI at atmospheric pressure is CH2OO (largely independent of pressure) and the other half or more is C4 sCI (consistent with Kroll OH yield measurements as well). To my eyes, this current work strongly confirms that prior finding, but if as claimed on lines 193-194 "Therefore, the
current dominant belief that in the isoprene ozonolysis sCIs at 760 Torr are mainly CH2OO, while [sic, with] no stabilized C4 CIs, is challenged." then in my opinion those holding the dominant belief have not properly read the literature.

In conclusion, I strongly favor publication, but I do feel nontrivial revisions in the discussion are required to put this extremely important work in the proper context.

The work as presented supports its claims clearly and convincingly, with sound methodology consistent with the highest standards. I am confident that the results could be reproduced by a careful experimentalist based on the description provided, which is detailed and precise.

Finally, most of the first paragraph of the discussion is a simple summary of the well written and concise presentation before hand. In my opinion this is superfluous and should be removed. There are also several typos or grammatical errors, which I point out in the attached annotated pdf.

Reviewer #3

(Remarks to the Author)
Dear Editor:

This manuscript reports the first direct measurement of Criegee intermediates (CIs) produced via the ozonolysis of isoprene---a critically important volatile organic compound (VOC). The authors employ UV cavity ringdown spectroscopy, a high-sensitivity technique, to achieve an excellent signal-to-noise ratio that allows for the unambiguous quantification of CH2OO.

The significance of this work has been highlighted by the authors in lines 50-55.
“Current experimental investigations of CIs rely on the surrogate generation technique by photolysis of diiodoalkanes in O2, but it does not replicate the energetic and chemical complexity of the alkene ozonolysis reaction network and cannot probe the nascent reaction dynamics and internal energy distributions of CIs. In addition, the yield of sCIs, their branching ratios, and major reaction pathways can only be determined in actual ozonolysis.”

Overall, this manuscript is clearly written; the experimental spectra are beautiful; it may be publishable after considering the following issues.

1 (minor).
The authors used SO2 titration (or scavenging) at a few places (examples copied below) to show that their signals are really from Criegee intermediates. This experimental validation is very important. However, their sentences misleadingly confuse Criegee intermediates with CH2OO. These MUST be corrected.

Lines 71-72: “The selective CI titrant, SO2, was added as an effective scavenger to further confirm that these features belong to CH2OO.”

Lines 110-111: “Thus, chemical titration strongly indicates the successful measurement of CH2OO in isoprene ozonolysis.”

Lines 103-105: “Further on, an excess amount of SO2 (9×1014 cm-3), which can effectively react with sCIs17,40,47,48 with a rate constant19,37 of 3.9 ×10−11 cm3 s−1, was added as a scavenger to confirm that the spectral features were from CH2OO.”

Lines 149-150: “The addition of the selective sCI titrant, SO2, served as an effective scavenger, further confirming these features as CH2OO.”

2 (minor).
The authors mentioned MACR absorption at a few places. How about MVK absorption? Given that MVK is one of the major products of isoprene ozonolysis, its potential interference should be addressed.

3 (major).
I find the discussion regarding pressure-dependent CI yields (Lines 161–186) difficult to follow and poorly supported by the presented data.

The experimental data in this study are limited to low pressure (7.5 Torr or 5–13 Torr), with the primary result being the 21% yield of stabilized CH2OO at 7.5 Torr (Line 161). While this is a significant finding, the authors provide no experimental data at higher pressures. Consequently, the discussion on pressure dependence relies almost entirely on external literature (e.g., Hakala et al., Ref. 53).

The current presentation is confusing because it may attribute conclusions about pressure dependence to this work when they are actually derived from others.

I suggest the authors:
•Refocus this section to emphasize how their low-pressure result compares with existing literature.
•Clearly distinguish between their own findings/analysis and previous reports.
•Ideally, perform additional experiments at higher pressures (approaching 760 Torr) to provide a firsthand account of the pressure dependence.

Version 1:

Reviewer comments:

Reviewer #1

(Remarks to the Author)
The authors have carefully addressed the concerns I expressed about the discussion of their results, and I believe that the revised manuscript will be a valuable contribution to our understanding of ozonolysis chemistry and I recommend its publication.

Reviewer #2

(Remarks to the Author)
In my opinion the authors have done an excellent job of responding to the reviewer comments, which were reasonably consistent with each other. The experimental finding is beautiful, compelling, and I believe it is a sufficient advance to warrant publication in Nature.

Reviewer #3

(Remarks to the Author)
Dear Editor:

Most of my concerns have been addressed in the revisions. One more point as below.

Lines 171-172: "In summary, this study shows that at low pressure, there are 21% stabilized CH2OO and no stabilized C4 CIs, while the remaining CIs consist of 37% hot CH2OO and 42% hot C4 CIs."

I guess that the 37% comes from 58% (value of Hakala et al.) minus 21% (this work) and that the 42% comes from 100% - 21% - 37%. However, I think that this is miss-interpreted and very misleading. The data of this work only support that "there is 21% stabilized CH2OO".

Open Access This Peer Review File is licensed under a Creative Commons Attribution 4.0 International License, which permits use, sharing, adaptation, distribution and reproduction in any medium or format, as long as you give appropriate credit to the original author(s) and the source, provide a link to the Creative Commons license, and indicate if changes were made.
In cases where reviewers are anonymous, credit should be given to 'Anonymous Referee' and the source.
The images or other third party material in this Peer Review File are included in the article's Creative Commons license, unless indicated otherwise in a credit line to the material. If material is not included in the article's Creative Commons license and your intended use is not permitted by statutory regulation or exceeds the permitted use, you will need to obtain permission directly from the copyright holder.
To view a copy of this license, visit https://creativecommons.org/licenses/by/4.0/

Response Letter of Manuscript ID NCOMMS-26-009340-T

Manuscript Title: "Direct measurement of Criegee intermediates in isoprene ozonolysis"

Point-by-point responses to the reviewers' comments are as follows.

We sincerely thank all the reviewers for careful evaluations of our manuscript and constructive and insightful comments. We have revised the manuscript in response to these comments to improve clarity and precision of interpretation. Point-by-point responses (blue text) to the reviewers' comments (replicated in black) are as follows, where the cited line numbers correspond to the track changes version of the manuscript.

Reviewer #1 (Remarks to the Author):

This work follows the direct detection of CH_2OO in ethene ozonolysis by this group last year, and now applies their technique to the atmospherically crucial reaction of ozone with isoprene. The experimental protocol is established and the measurement of CH_2OO yield is rigorous and described clearly. The measurements directly quantify the CH_2OO and place an upper limit on the other possible carbonyl oxide products at low pressure -- that is the extent of the new experimental data.

The implications of the work, however, rely on combination of this measurement with the body of literature data to infer that the atmospheric pressure reaction may have a larger contribution of C_4 carbonyl oxides than some models have acknowledged. The role of the present measurement in establishing, rather than merely corroborating, that conclusion should be described more clearly. In addition, the appearance of a mass spectrometric determination of C_4 carbonyl oxide yields from atmospheric pressure isoprene ozonolysis (ACS Earth Space Chem. 2026, 10, 2, 328–337) changes the environment for the present work (that paper probably appeared around the time this work was submitted).

From the yields implied by the product measurements (e.g., in Supplemental Table 2), the discrepancy to be addressed is the contrast between the CH_2OO specific (like HMHP measurements) that suggest CH_2OO of about 30% and the predictions of the Nguyen et al. model, which interpreted the ~60% carbonyl oxide yield as being dominated by CH_2OO, based on comprehensive product characterization. However, several other chamber studies (ref. 40 and 52, for example) suggested the presence of C_4 carbonyl oxides. Hakala and Donohue proposed a model that bridged these apparent discrepancies, predicting a weakly pressure dependent yield of stabilized CH_2OO at ~30%, with the rest of the Nguyen et al yield attributed to C_4 species. I give that background, which is in the manuscript but in a kind of confusing way, to point out that the overall picture that this work fits into had been coming together already. In that context, this measurement confirms one leg of the Hakala and Donohue proposal, of a weakly pressure-dependent stable CH_2OO yield, and the Chhantyl-Pun et al. paper that recently appeared (ACS Earth Space Chem. 2026, 10, 2, 328–337), confirms the other, a significant (~11%) yield of stabilized C_4 carbonyl oxides. As a result, and given that the C_4 yield measurement has just appeared, I recommend that the authors reconsider their discussion section; first I am not sure how

"dominant" was the belief that CH_2OO is the only stabilized carbonyl oxide in atmospheric pressure ozonolysis, and second because with the other recent result this provides direct-detection confirmation of a framework that could unify the picture of carbonyl oxide formation in isoprene ozonolysis.

Response:

We thank the reviewer for the thoughtful and detailed comments and for recognizing the rigor of the experimental measurements. Following the reviewer's recommendation to reconsider the discussion section, we have revised the discussion in Lines 165-200.

Specifically, regarding the reviewer's first comment on how "dominant" was the belief that CH_2OO is the only stabilized carbonyl oxide in atmospheric pressure ozonolysis, we have removed the language suggesting that CH_2OO was largely considered the sole stabilized carbonyl oxide at atmospheric pressure. The revised discussion now presents our results as providing direct experimental confirmation of the low-pressure boundary of the emerging framework, rather than as challenging a prevailing consensus.

Regarding the reviewer's second comment that, with the other recent result (Chhantyl-Pun *et al.* *ACS Earth Space Chem*. 2026, 10, 328–337), our current study provides direct-detection confirmation of a framework that could unify the picture of carbonyl oxide formation in isoprene ozonolysis, we agree that the contextual framing of the pressure-dependent interpretation of yields requires clarification. In the revised discussion in Lines 165-200, we made it clearer to distinguish between the direct experimental findings of the present study and interpretations derived from prior literature. Specifically, we emphasize that our low-pressure measurement of a ~21% stabilized CH_2OO yield provides directly the yields and types of nascent sCIs in the net isoprene + ozone reaction (prior to any pressure stabilization of CIs), i.e., the lower boundary, and direct confirmation of the weak pressure dependence proposed in previous modeling studies (e.g., Hakala and Donohue), rather than presenting this as a reinterpretation of atmospheric-pressure yields. Furthermore, we appreciate the reviewer's note regarding the recent study by Chhantyl-Pun *et al.* (*ACS Earth Space Chem*. 2026, 10, 328–337). At the time of our original submission, that work had not been published. We have now added the citation to that paper in our discussion in Lines 191-193, "A recent atmospheric-pressure study further reported ~11% C_4 sCIs formed in isoprene ozonolysis[58], supporting the presence of non-negligible C_4 sCIs contributions". Nevertheless, we would like to point out that the recent work by Chhantyl-Pun *et al.* stated in the introduction, "previous studies underestimate the potential of larger sCIs to participate in bimolecular processes in the atmosphere", and that that study was based on indirect measurements of sCIs at atmospheric pressure, rather than direct detection of sCIs in our study.

Reviewer #2 (Remarks to the Author):

This work claims, as far as I know correctly, to be the first direct measurement of carbonyl oxides from isoprene ozonolysis. It is a very careful experimental study in a low-pressure (7 torr) flow cell using UV CRDS spectroscopy, including a thorough kinetic analysis as well as complementary experiments with an sCI selective scavenger (SO_2) to confirm the observed signals are consistent with a carbonyl oxide - specifically formaldehyde oxide, CH_2OO.

The results will be broadly interesting to both the physical chemistry and atmospheric chemistry communities. Direct observation of sCI from ozonolysis has been extremely difficult, as ozonolysis produces a rich set of products quite quickly, and the sCI concentrations are often very low (and they are very short lived). They however play an important role in the atmosphere, where atmospheric modeling (and field observations) have shown that sCI + SO_2 can be a significant source of gas-phase H_2SO_4 and so an important contributor to atmospheric new-particle formation.

I believe that the core finding - experimental confirmation of a 20% sCI yield at 7 torr, consisting almost entirely of CH_2OO - is of sufficiently high interest to merit publication in Nature Communications. It is also my opinion that the core of the manuscript as presented, along with the supplemental information, presents this case clearly.

Response: We thank the reviewer for recognizing the rigor of the experimental approach and the significance of the direct measurement of stabilized Criegee intermediates in isoprene ozonolysis, and for thoughtful feedback that helps refine the framing and clarity of the manuscript.

However, I do have some reservations about the discussion and the context, which in my opinion are in some places an oversell. Specifically:

1. In my opinion there was very strong evidence that CIs form in atmospheric isoprene ozonolysis. I would have been absolutely stunned if a paper appeared claiming this was not so. Claiming this as a fundamental "unresolved" question thus strikes me as too strong an assertion. A major reason is that, not only is there strong evidence from scavenger studies (not just in environmental chambers, but also pressure-dependent flow tubes in the cited Hakala paper), but complementary results, not discussed in this paper, addressing OH yields from isoprene ozonolysis are also strongly consistent with CI production. Specifically, Kroll et al GRL 2001 described OH formation measurements using a highly accurate atmospheric laser induced fluorescence instrument, showing a modestly decreasing OH yield from about 20% at 10 torr to about 10% at 40 torr. This is consistent with OH formation largely by excited C4 CI starting to be stablized over this range.

that brings me to

2. The discussion as worded suggests that a significant role for C4 sCI approaching atmospheric pressure is a new interpretation for this manuscript. I find that surprising, as this is exactly the argument made in the Hakala paper, which concludes that about half of the sCI at atmospheric pressure is CH_2OO (largely independent of pressure) and the other half or more is C_4 sCI (consistent with Kroll OH yield measurements as well). To my eyes, this current work strongly confirms that prior finding, but if as claimed on lines 193-194 "Therefore, the current dominant

belief that in the isoprene ozonolysis sCIs at 760 Torr are mainly CH_2OO, while [sic, with] no stabilized C_4 CIs, is challenged." then in my opinion those holding the dominant belief have not properly read the literature.

In conclusion, I strongly favor publication, but I do feel nontrivial revisions in the discussion are required to put this extremely important work in the proper context.

Response: We have removed all the statements in questions. Specifically, for (1), "do CIs actually form in atmospheric isoprene ozonolysis" and (2) "C_4 sCIs can play a much more important role than previously thought".

(1) We agree that substantial indirect evidence has supported CI formation in isoprene ozonolysis, including scavenger-based chamber studies, pressure-dependent flow tube experiments, and complementary OH yield measurements. Our original wording may have overstated the degree of uncertainty in the field. To address this concern, we have removed that statement and revised the manuscript in Lines 57-61: "some fundamental questions remain regarding their direct characterization in ozonolysis reactions, their lifetimes under atmospheric conditions, and the extent to which they influence tropospheric chemistry." Overall, the revised introduction here avoids implying that CI formation itself was doubted; instead, the introduction now only clarifies that direct observation of any CI from isoprene ozonolysis had not been achieved previously. We also added citation to the OH-yield-measurement work by Kroll et al. (GRL 2001) as reference [28].

(2) We also agree that the possibility of significant C_4 stabilized CI formation at atmospheric pressure has been proposed previously, particularly in the work of Hakala and Donohue. Our intention was not to present this as a new interpretation, but rather to provide direct experimental confirmation of key elements of that framework through independent and direct measurement. To avoid any implication that we were challenging a "dominant belief," we have removed this and revised the sentences (in Lines 20, 84, 171-172, 208-209, and 219-220), which previously implied that CH_2OO was widely considered the only stabilized carbonyl oxide at atmospheric pressure. The discussions of this part have been substantially revised to clearly differentiate direct findings of this work and those of previous studies, in Lines 165-200.

The work as presented supports its claims clearly and convincingly, with sound methodology consistent with the highest standards. I am confident that the results could be reproduced by a careful experimentalist based on the description provided, which is detailed and precise.

Finally, most of the first paragraph of the discussion is a simple summary of the well written and concise presentation before hand. In my opinion this is superfluous and should be removed. There are also several typos or grammatical errors, which I point out in the attached annotated pdf.

Response: In accordance with the reviewer's suggestion, we have removed the first paragraph of the discussion (Lines 149-159) that redundantly summarizes the results section. The revised discussion is now more concise and focused on interpretation and contextualization.

All typographical and grammatical errors identified in the annotated PDF have been corrected.

Reviewer #3 (Remarks to the Author):

Dear Editor:

This manuscript reports the first direct measurement of Criegee intermediates (CIs) produced via the ozonolysis of isoprene---a critically important volatile organic compound (VOC). The authors employ UV cavity ringdown spectroscopy, a high-sensitivity technique, to achieve an excellent signal-to-noise ratio that allows for the unambiguous quantification of CH_2OO.

The significance of this work has been highlighted by the authors in lines 50-55.
"Current experimental investigations of CIs rely on the surrogate generation technique by photolysis of diiodoalkanes in O_2, but it does not replicate the energetic and chemical complexity of the alkene ozonolysis reaction network and cannot probe the nascent reaction dynamics and internal energy distributions of CIs. In addition, the yield of sCIs, their branching ratios, and major reaction pathways can only be determined in actual ozonolysis."

Overall, this manuscript is clearly written; the experimental spectra are beautiful; it may be publishable after considering the following issues.

Response: We thank the reviewer for the positive assessment of the experimental approach and spectral quality and for the constructive suggestions provided below.

Comment 1 (minor).
The authors used SO_2 titration (or scavenging) at a few places (examples copied below) to show that their signals are really from Criegee intermediates. This experimental validation is very important. However, their sentences misleadingly confuse Criegee intermediates with CH_2OO. These MUST be corrected.

Lines 71-72: "The selective CI titrant, SO_2, was added as an effective scavenger to further confirm that these features belong to **CH_2OO**."

Lines 110-111: "Thus, chemical titration strongly indicates the successful measurement of **CH_2OO** in isoprene ozonolysis."

Lines 103-105: "Further on, an excess amount of SO_2 (9×10^{14} cm^{-3}), which can effectively react with sCIs[17,40,47,48] with a rate constant[19,37] of 3.9×10^{-11} cm^3 s^{-1}, was added as a scavenger to confirm that the spectral features were from **CH_2OO**."

Lines 149-150: "The addition of the selective sCI titrant, SO_2, served as an effective scavenger, further confirming these features as **CH_2OO**."

Response: We agree that the SO_2 titration experiment confirms the presence of Criegee intermediates (CIs) more generally than just CH_2OO and should not be described in a way that only specifies for CH_2OO (although in this study of low-pressure isoprene ozonolysis CH_2OO was

found to be essentially the only sCI). Accordingly, we have revised these "**CH_2OO**" to "**CIs**", to eliminate any potential ambiguity (Lines 74, 108, and 113).

Comment 2 (minor).

The authors mentioned MACR absorption at a few places. How about MVK absorption? Given that MVK is one of the major products of isoprene ozonolysis, its potential interference should be addressed.

Response: In the spectral fitting procedure shown in Fig. 2, the potential contributions of all relevant carbonyl species (MVK, MACR, HCHO), carbonyl oxides (CH_2OO, MVK-oxide, MACR-oxide), and ozone were considered. MVK does not exhibit sharp absorption features in the 363-394 nm range and has small cross sections (< 3×10^{-20} cm^2) and low yield (~17%) compared to MACR (41%) or HCHO (42%) in isoprene ozonolysis; HCHO has a relatively narrower feature at 370 nm but even smaller cross sections (< 1×10^{-21} cm^2); cross sections of ozone are < 5×10^{-23} cm^2 in this range. Therefore, their contributions to the absorption spectra in the 363-394 nm range are much smaller than MACR and not discernible. This clarification is now added in the caption of Fig. 2, "Contributions from MVK, HCHO, and O_3 (or other species) are not included in the fitting because their absorptions in the 363–394 nm range are small (less than MACR) and not discernible." In addition, because the CI concentrations in Fig. 4 were determined from the difference spectra between "ozonolysis" and "ozonolysis + SO_2" (see Supplementary Figures 5–7), the carbonyl contributions were already removed from the spectra and did not affect CI kinetic results. This clarification was in Lines 121-124 and has now also been added to the caption of Figure 4, "[CH_2OO]s are determined from difference spectra between ozonolysis and ozonolysis + SO_2 conditions (as detailed in Supplementary Fig. 5-7), which remove potential UV absorption contributions from carbonyls, O_3, and other broad background."

Comment 3 (major).

I find the discussion regarding pressure-dependent CI yields (Lines 161–186) difficult to follow and poorly supported by the presented data.

The experimental data in this study are limited to low pressure (7.5 Torr or 5–13 Torr), with the primary result being the 21% yield of stabilized CH_2OO at 7.5 Torr (Line 161). While this is a significant finding, the authors provide no experimental data at higher pressures. Consequently, the discussion on pressure dependence relies almost entirely on external literature (e.g., Hakala et al., Ref. 53).

The current presentation is confusing because it may attribute conclusions about pressure dependence to this work when they are actually derived from others.

I suggest the authors:

•Refocus this section to emphasize how their low-pressure result compares with existing literature.

•Clearly distinguish between their own findings/analysis and previous reports.

•Ideally, perform additional experiments at higher pressures (approaching 760 Torr) to provide a firsthand account of the pressure dependence.

Response: We agree that the original discussion section did not sufficiently distinguish between the direct experimental findings of the present work and pressure-dependent interpretations drawn from previous studies. To address this concern, we have substantially revised this section in Lines 163-200.

Specifically, we now clearly state in Lines 163, 168-169, and 193-195 that our direct experimental measurements are in the low-pressure regime (5–13 Torr). We emphasize that our low-pressure measurement of a ~21% stabilized CH_2OO yield provides directly the yields and types of nascent sCIs in the net isoprene + ozone reaction (prior to any pressure stabilization of CIs), i.e., the lower boundary, and direct confirmation of the weak pressure dependence proposed in previous modeling studies (e.g., Hakala and Donohue), rather than presenting this as a reinterpretation of atmospheric-pressure yields. We have revised the language to avoid implying that the conclusions regarding atmospheric-pressure stabilization originate from the present measurements. For example, in Lines 195-200, "At 760 Torr, previous studies indicate that the total sCI yield increases to ~61%, reflecting additional stabilization of both CH_2OO and C_4 CIs due to collisions."

Regarding higher-pressure experiments, the current flow-cell configuration is optimized for maintaining short residence times required for resolving nascent intermediates at low pressure. Extension to higher pressures would require modifications to maintain comparable residence time (and could be performed in future studies). We have clarified this point in the revised manuscript in Lines 162-164: "The measurements of sCIs in this study were performed at 5–13 Torr but could be extended to higher pressure when a higher pumping speed is available to maintain the short residence time."

Response Letter of Manuscript ID NCOMMS-26-009340A

Manuscript Title: "Direct measurement of Criegee intermediates in isoprene ozonolysis"

We sincerely thank all reviewers for their thoughtful and positive evaluations of this work.

We have addressed the remaining comment of reviewer #3 and completed all editorial revisions as requested.

Reviewer #1 (Remarks to the Author):

The authors have carefully addressed the concerns I expressed about the discussion of their results, and I believe that the revised manuscript will be a valuable contribution to our understanding of ozonolysis chemistry and I recommend its publication.

Reviewer #2 (Remarks to the Author):

In my opinion the authors have done an excellent job of responding to the reviewer comments, which were reasonably consistent with each other. The experimental finding is beautiful, compelling, and I believe it is a sufficient advance to warrant publication in *Nature*.

Response to Reviewer #3

Most of my concerns have been addressed in the revisions. One more point as below.

Lines 171-172: "In summary, this study shows that at low pressure, there are 21% stabilized CH_2OO and no stabilized C4 CIs, while the remaining CIs consist of 37% hot CH_2OO and 42% hot C4 CIs."

I guess that the 37% comes from 58% (value of Hakala et al.) minus 21% (this work) and that the 42% comes from 100% - 21% - 37%. However, I think that this is miss-interpreted and very misleading. The data of this work only support that "there is 21% stabilized CH_2OO".

Response: Thank you for the comment that indicates a need for clarificaiton. These values are in fact all derived from and supported by the data of this work. Note that besides Criegee intermediates, this work also performed measurements of HCHO, O_3, and MACR. Although the "37% hot CH_2OO" and "42% hot C4 CIs" are not from direct measurements of hot Criegee intermediates, they are still based on our experimental results (see lines 127-129, 137-144, 241-246, Supplementary Fig. 8 and Supplementary Table 1). To clarify, lines 171-172 have been revised and are replicated here: "In summary, this study shows that at low pressure, there are 21% stabilized CH_2OO and no stabilized C_4 CIs (from direct experimental measurements of CIs), while the remaining CIs are estimated to consist of 37% hot CH_2OO and 42% hot C_4 CIs (based on kinetic simulations and experimental constrains of HCHO, O_3 and MACR, as detailed in Supplementary Table 1 and Methods)." Also added as a clarification in lines 243-248: "The total CH_2OO yield, which is complementary to the primary HCHO yield, was constrained by combining time-resolved measurements of HCHO and O_3 with kinetic simulations, in which secondary

HCHO formation pathways were explicitly accounted for (see Supplementary Table 1). MACR measurements provided an additional constraint on product branching. Branching ratios of all the primary pathways of isoprene ozonolysis were established (see Supplementary Table 1)."

A more detailed explanation is provided as follows. The total yield of CH_2OO (58%, including 21% stabilized CH_2OO + 37% hot CH_2OO) is constrained experimentally through measurements of formaldehyde (HCHO), which is formed as coproducts with C4 CIs in the complementary pathways in ozonolysis, such that the total CH_2OO yield can be approximated to be 100% minus the primary HCHO yield (see Fig. 1 and reference [17]). However, secondary reactions of Criegee intermediate (e.g. CH_2OO + O_3, CH_2OO + isoprene, and more in Supplementary Table 1) introduce a small amount secondary HCHO production; if the experimentally measured [HCHO] is used directly for the primary HCHO yield, there would be a small systematic overestimation. Therefore, instead of relying on a simple ratio of measured concentrations of $[HCHO]_i/\Delta[O_3]$, we combine time-resolved measurements of HCHO and O_3 with kinetic simulations to account for secondary HCHO formation and reaction pathways and arrive at accurately the primary HCHO yield. This gives the total yield of CH_2OO (58%, which is further quantified to be 21% stabilized CH_2OO and 37% hot CH_2OO by the CI measurements here) and by balancing the total yield of C4 CIs (42%, which is further quantified to be 0% stabilized C4 CIs and 42% hot C4 CIs by the CI measurements). In addition, the MACR measurements provide a complementary constraint on the CH_2OO + MACR branching channel, such that, together with HCHO (and assuming HCHO + MACR + MVK ≈ 100% yield as in previous studies), all primary pathways are internally constrained in this study. This approach follows the same methodology in our previous work in reference [48-49], where secondary contributions of HCHO were explicitly quantified and corrected. Therefore, the inferred total CH_2OO yield and the partitioning into hot CIs are based on our kinetic simulation with experimental constraints in this work, rather than simply coming from literature values. These results are consistent with prior studies, which also used carbonyl yields to obtain CH_2OO vs C4 CI branchings, such as Nguyen et al. in reference [17] in line 236.
